# A systematic approach to quantify the influence of indoor environmental parameters on students' perceptions, responses, and short‐term academic performance

**DOI:** 10.1111/ina.13116

**Published:** 2022-10-17

**Authors:** Henk W. Brink, Marcel G. L. C. Loomans, Mark P. Mobach, Helianthe S. M. Kort

**Affiliations:** ^1^ Research Group Facility Management, Research Centre for Built Environment NoorderRuimte Hanze University of Applied Sciences Groningen The Netherlands; ^2^ Department of the Built Environment, Building Performance IEQ‐Health Eindhoven University of Technology Eindhoven The Netherlands; ^3^ Research Group Spatial Environment and the User, Research Centre Mission Zero The Hague University of Applied Sciences The Hague The Netherlands; ^4^ Research Group Technology for Healthcare Innovations, Research Centre Sustainable and Healthy Living Utrecht University of Applied Sciences Utrecht The Netherlands

**Keywords:** performance, classrooms' indoor environmental quality, health, higher education, student responses, quality of learning

## Abstract

Several studies found that classrooms' indoor environmental quality (IEQ) can positively influence in‐class activities. Understanding and quantifying the combined effect of four indoor environmental parameters, namely indoor air quality and thermal, acoustic, and lighting conditions on people is essential to create an optimal IEQ. Accordingly, a systematic approach was developed to study the effect of multiple IEQ parameters simultaneously. Methods for measuring the IEQ and students' perceived IEQ, internal responses, and academic performance were derived from literature. Next, this systematic approach was tested in a pilot study during a regular academic course. The perceptions, internal responses, and short‐term academic performance of participating students (*n* = 163) were measured. During the pilot study, the IEQ of the classrooms varied slightly. Significant associations (*p* < 0.05) were observed between these natural variations and students' perceptions of the thermal environment and indoor air quality. These perceptions were significantly associated with their physiological and cognitive responses (*p* < 0.05). Furthermore, students' perceived cognitive responses were associated with their short‐term academic performance (*p* < 0.01). The observed associations confirm the construct validity of the systematic approach. However, its validity for investigating the influence of lighting remains to be determined.

**Practical implications**

- Application of this systematic approach allows measurement of the combined effect of four indoor environmental quality (IEQ) parameters, namely indoor air quality and thermal, acoustic, and lighting conditions, on short‐term academic performance simultaneously.
- Natural variations in the IEQ were observed during regular academic courses, which significantly influenced students' IEQ perceptions.
- Students' IEQ perceptions were significantly associated with their perceived cognitive performance.
- Students' cognitive performance was significantly associated with their short‐term academic performance, but explained only a small amount of variance of short‐term academic performance.

## 1 INTRODUCTION

This study explores how to measure the influence of the indoor environmental quality (IEQ) parameters on students and their academic performance. It is the primary responsibility of the school management to provide appropriate classrooms for education; which can positively influence students' academic performance, contributing to a sustainable and positive school climate.^1^ As part of classrooms' environmental quality, this study focusses on four IEQ parameters: (1) indoor air quality, (2) thermal conditions, (3) acoustic conditions, and (4) lighting conditions.^2^ This study examines how to measure the combined influence of all four IEQ parameters on the academic performance of students in higher education. The academic performance of students is acknowledged as an important study outcome, besides behavioral and psychological outcomes.^3^

In the last decade, there has been an increasing interest in developing a more holistic approach for examining the influence of IEQ conditions on students' academic performance.^4^ Previous research on the combined influence of two or more IEQ parameters found that IEQ does influence students' performance.^4^ For example, Wargocki and Wyon^5^ demonstrated how cognitive performance is influenced by thermal conditions and indoor air quality. Other studies have examined the combined influence of thermal conditions and indoor air quality.^6^, ^7^ These studies show that poor IEQ affects students' cognitive performance in higher education. Xiong et al.,^8^ who explored the impacts of three IEQ parameters, namely thermal, acoustic, and visual conditions on students' cognitive performance, concluded that optimal IEQ conditions in which students perform at their best, are task‐dependent, with students preferring a relatively cool, bright, and quiet environment. However, few studies have examined the combined influence of all four IEQ parameters.^4^

A holistic assessment of indoor environmental conditions is important because of the mutual interaction of IEQ parameters. This interaction was observed by Kim and De Dear,^9^ who developed a model to determine these interaction effects and the existence of a hierarchy among IEQ parameters in another setting. Two basic IEQ factors, namely temperature and noise level, were identified on the basis of data collected in office environments. The negative impact of these factors outweighs their positive effects on the overall experience of IEQ. Air quality, the amount of light in the workplace, visual comfort related to the lighting, and sound privacy were classified as proportional IEQ factors. The overall occupant satisfaction increased or decreased in linear proportion to the building's performance impacting these factors.^9^

**FIGURE 1:**
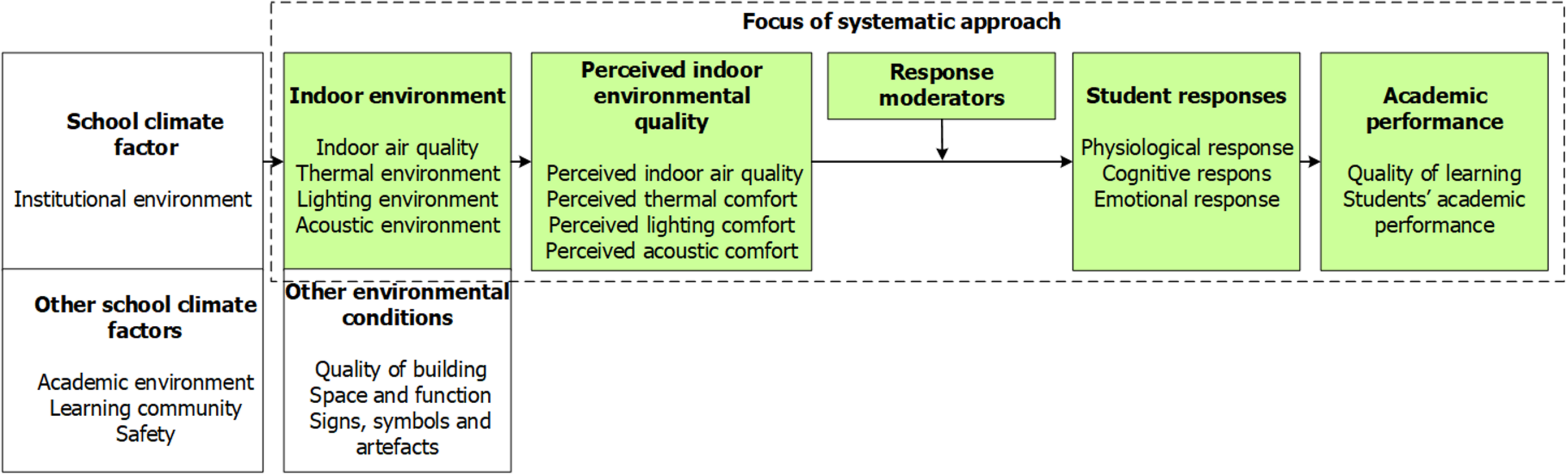
The systematic approach, based on Wang and Degol^3^ and Bitner^11^

Although previous studies have explored the influence of the above‐mentioned parameters, to the best of our knowledge, no study has combined the four separate parameters within a systematic approach to examine the impacts of the IEQ in higher education classrooms. Therefore, there is a need to develop models for assessing the influence of multiple environmental parameters on students' performance.^10^ To assess this influence, a framework of Bitner^11^ is used. This framework was selected because it addresses the combined influence of different environmental factors, including all four IEQ parameters. To enable its application in higher education classrooms, the relationships described in the work of Wang and Degol^3^ were fitted into this framework. Figure 1 presents the framework for understanding IEQ‐user relationships in classrooms and outlines the systematic approach.

The main objective of this study was to develop and validate a systematic approach for measuring the effect of all four IEQ parameters in higher education classroom on students' perceptions, responses, and academic performance. The application of this systematic approach benefit future studies seeking to determine the influence of both single and multiple IEQ parameters. Furthermore, it will be possible to determine whether there is a hierarchy between IEQ parameters in assessment of the impacts of IEQ on users in higher education classrooms. In this work, existing methods are used for measuring the influence of IEQ parameters on students and their academic performance. Subsequently, a pilot study is conducted to assess the validity and applicability of the systematic approach in real‐life conditions. In the next section, the development of the systematic approach is described, followed by its application in the third section.

## 2 A SYSTEMATIC APPROACH FOR ASSESSING THE EFFECTS OF MULTIPLE IEQ PARAMETERS

### 2.1 Method

The development of a systematic approach entailed the following three phases: (1) compilation of available information on how to measure IEQ and higher education students' perceptions, responses, and academic performance, (2) categorization of the available information on these methods, and (3) adjustments of the identified methods and tests if needed and their incorporation into the systematic approach. This paragraph presents an overview of these three phases. Appendix A provides a list of the nomenclature for indicators of the IEQ with abbreviations.

During the first phase, available information on how to measure the IEQ and the influence of the IEQ on students' perceptions, responses and academic performance was collected from the literature. Potentially relevant publications were identified through searches in the following databases: Web of Science, Scopus, Emerald Insight, Wiley Online Library, Sage, PubMed, and 27 EBSCOhost databases (e.g., Academic Search Premier, ERIC, APA PsycINFO, Teacher Reference Center). For the search, keywords relating to classrooms' IEQ, teaching and learning, and students' academic performance were used. Publications whose titles, keywords or abstracts did not indicate that indoor environmental conditions were the topic of study were excluded (*n* = 1162). These publications emerged in the primary search because one or more keywords were used in different contexts. Publications that only addressed physical indoor environmental conditions or other types of building performance (e.g., energy consumption and sustainability) and did not analyze their effects on teaching, learning or academic performance were excluded (*n* = 102). Finally, publications were excluded that addressed people with physical or mental disabilities (*n* = 23), those that did not address classrooms in higher education (*n* = 54) and those that were not written in English (*n* = 3).

**FIGURE 2:**
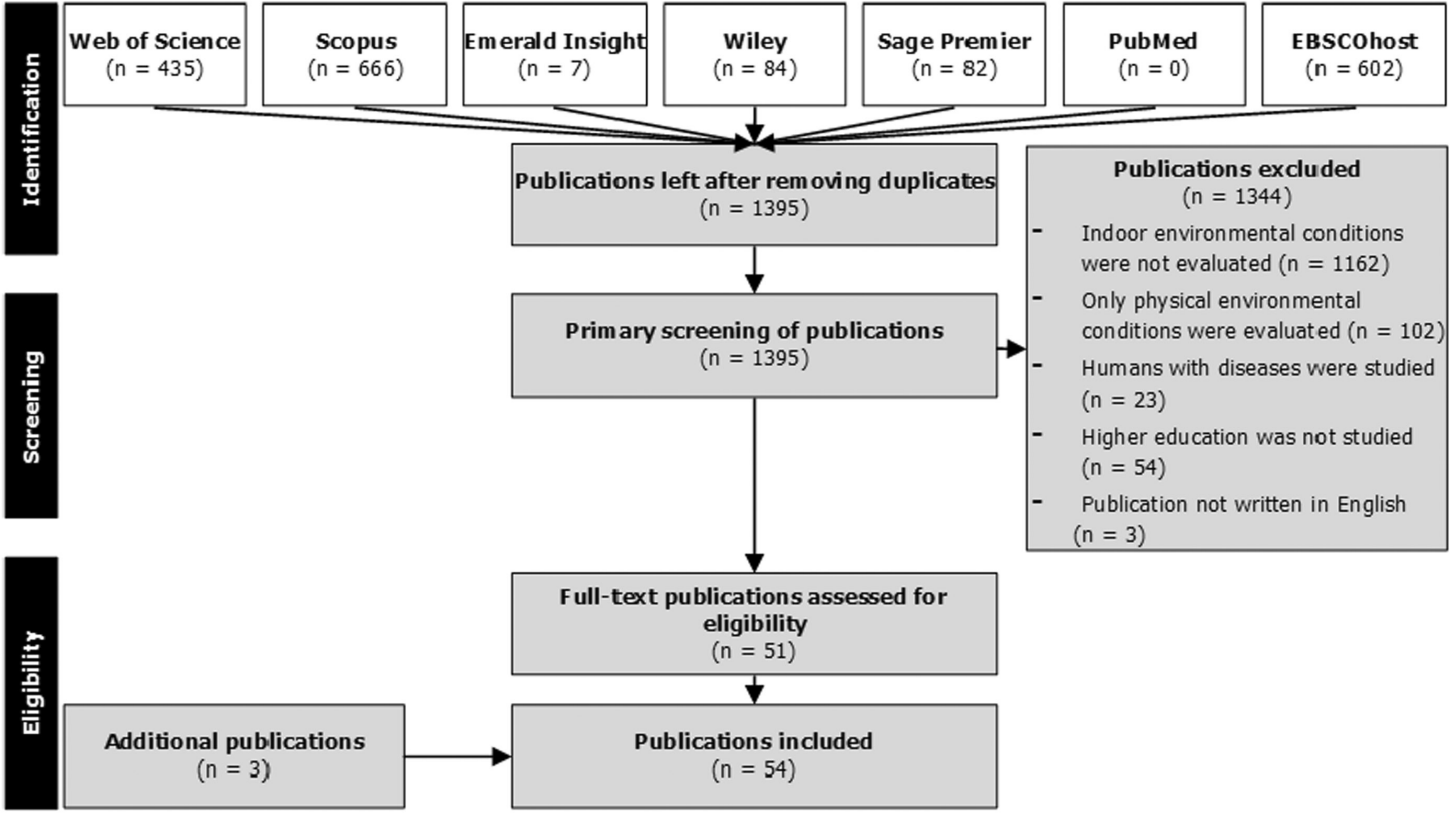
Screening process of literature for systematic approach development

Following this selection stage, 51 publications were included, to which three additional publications were added.^12^, ^13^, ^14^ These additional publications were cited by resp. Castilla et al.,^15^ Corgnati et al.,^16^ and De Abreu‐Harbich et al.^17^ and provided relevant additional information about the applied methods. In place of a study by Kooi et al.,^4^, ^18^ the more complete publication by Mishra et al.^19^ on the same study was used. Further details on the applied search string and exclusion criteria can be found in a previous systematic literature review,^4^ which provides an overview of how all four IEQ parameters influence students' perceptions, responses, and academic performance. Figure 2 shows the outcome of the selection stages during the screening of the identified publications.

In the second phase of the systematic approach development, the available information on the applied methods was categorized, according the categories in Figure 1. In addition, the available questionnaires in the corresponding manuscripts were arranged by topic, for example all items which address the perceived indoor air quality or thermal comfort.

During the third and final phase, constraints were set for applying the systematic approach. Methods and tests were used to compose the approach which showed statistically significant associations between the short‐term influence of the IEQ on students' perceptions, responses, and academic performance. If necessary, items, addressing the perceived IEQ, students' perceived cognitive responses to the IEQ, and students' perceived academic performance were reformulated to enable the use of a single, uniform response scale. Three experts from professional and education fields, who deal with indoor environment issues on a daily basis, were consulted to assess the content and face validity of the composed questionnaire. The consulted experts were a senior lecturer and researcher, who specializes in building physics, of The Hague University of Applied Sciences, a consultant focusing on sustainability and health from DGMR Advisors for Construction, Industry, Traffic, and Environment, and an advisor on indoor climate control from Nijeboer‐Hage Technical Advisors, all of whom are located in the Netherlands. As a final step to enable the application of the systematic approach in higher education Dutch‐language classes, the composed questionnaire was translated into Dutch with the help of a bilingual expert.

### 2.2 Outcome of the process of developing a systematic approach

In this subsection, the results are presented, per category, of all the phases of development of the systematic approach according to the framework shown in Figure 1. First, the identified IEQ indicators are presented for determining the actual IEQ, followed by a description of the methods used to measure students' perceptions of the IEQ, their internal responses, and their academic performance. Lastly, the fully composed systematic approach is presented. Appendix B presents an overview of all included empirical studies and those used for developing the approach. It also lists all of the indicators used to measure the IEQ and presents detailed information on methods for measuring students' perceptions, responses, and academic performance.

#### 2.2.1 Indoor environment

**FIGURE 3:**
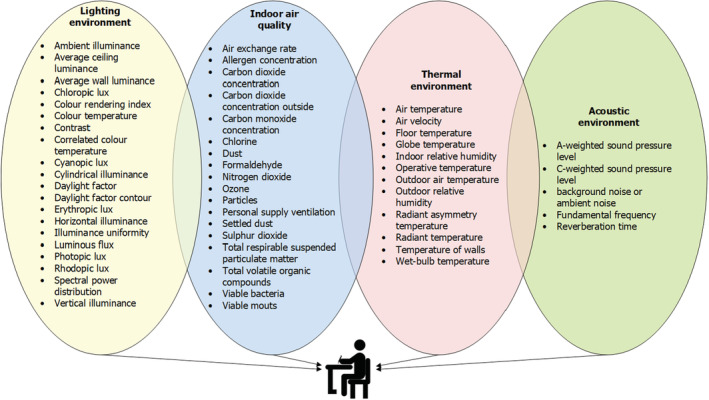
Indoor environmental quality indicators grouped by category

It is essential to measure specific IEQ parameters to determine the quality of the indoor environment. With reference to the available information in the selected publications, 54 indicators were identified, which reflect the quality of the four indoor environmental parameters. Figure 3 presents these indicators, grouped by IEQ parameters.

#### 2.2.2 Perceived indoor environmental quality

**FIGURE 4:**
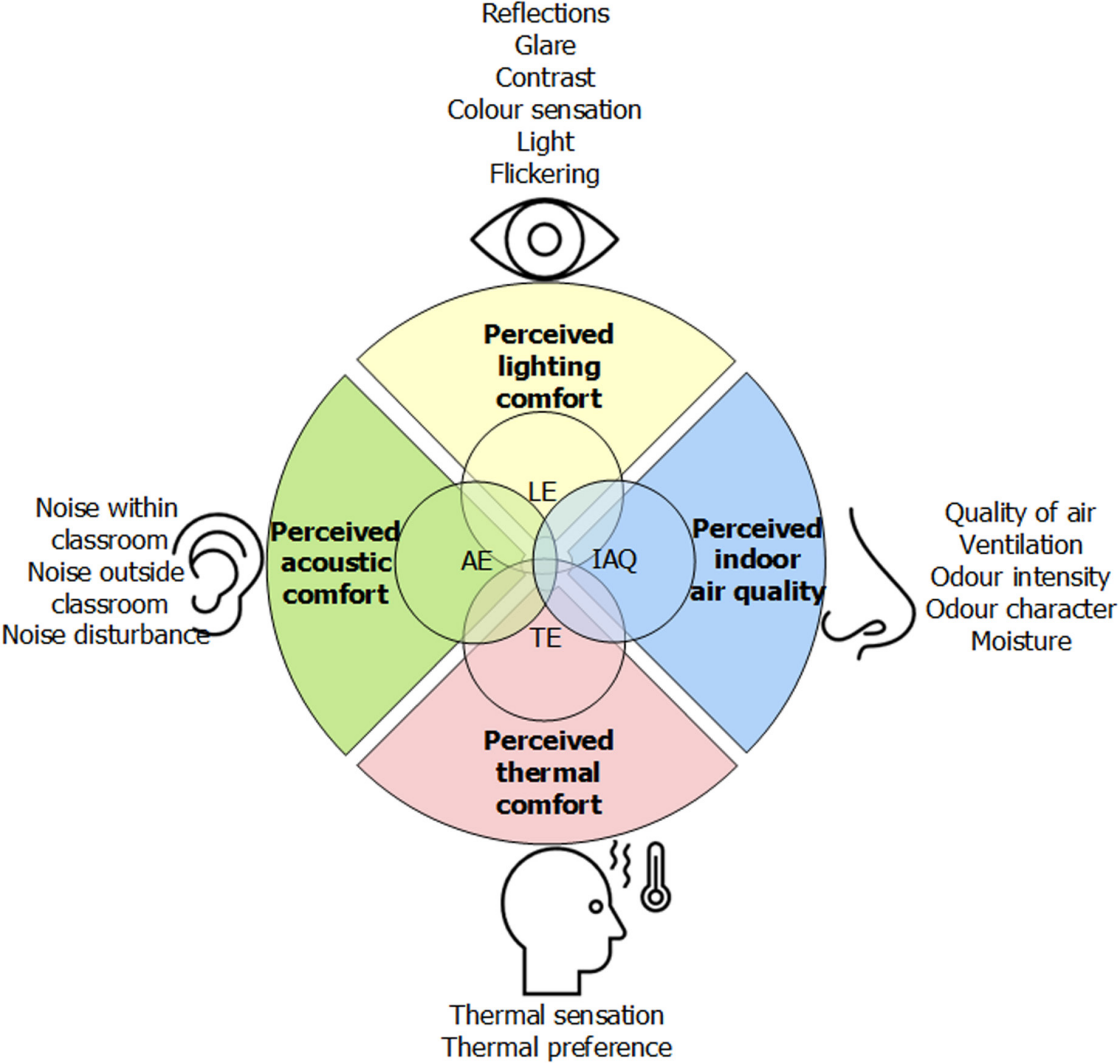
Perceived indoor environmental quality categories and subcategories. LE,Lighting environment; IAQ,Indoor air quality; TE,Thermal environment; AE,Acoustic environment

Respondents in the reviewed studies responded to items for measuring the perceived quality of the indoor environment. Available information on measurement of the perceived IEQ presented in text and questionnaires included in the reviewed studies were grouped to each specific IEQ parameter. Subsequently, 16 subcategories were identified for measuring the perceived IEQ according to the topics covered by the questionnaire developed for implementing the systematic approach. The questionnaire with these subcategories was then validated. Figure 4 presents the subcategories and their relations to specific indoor environmental parameters.

The response options applied in the identified studies were analyzed to develop standard response options for use in the systematic approach. The most frequently used scale was a 5‐point Likert scale, ranging from 1 (*disagree*) to 5 (*agree*).^20^, ^21^, ^22^, ^23^, ^24^ Therefore, this scale was adopted for the questionnaire. In some cases, however, items had to be reformulated, so they could be answered with this scale. A senior lecturer and researcher of The Hague University of Applied Sciences assisted the authors in this critical process. For example, the topic of dry air was addressed by Yang, Becerik‐Gerber and Mino.^25^ In one study,^26^ respondents were asked to assess the air humidity and in another study,^24^ respondents described how they felt about the degree of humidity. Accordingly, in the questionnaire, the topic of air moisture is addressed with the following reformulated item “The air is dry in here.” The questionnaire was then assessed by the above‐described experts for relevance and applicability in relation to higher education classrooms. This evaluation process led to the deletion of two items. The first item, “The illumination provided by artificial sources in the classroom compared to the shape of the classroom itself (geometry of the classroom) is inadequate”,^13^ was deleted because an expert on the topic indicated that this question was too difficult to understand. The second item, “The light seeping through windows appears to be inadequate”,^13^ was deleted because an expert on the topic indicated that this question is not valid because there is always a combination of daylight and artificial light in the classroom, so the amount of daylight cannot be assessed by the respondent.

After data‐collection, mean scores were calculated for each perceived IEQ scale for further analysis. The lowest average perception score, derived from individuals' scores, was 1 (very poor); the maximum perception score is 5 (very good). When assessing perceived thermal comfort, it is necessary to include thermal acclimation, defined as the adaptive changes that occur within individuals,^27^ because it may influence the actual thermal sensation, especially within the first 20 min after entering a classroom.^19^ In addition, the amount of clothing expressed as an individual's clothing insulation value, could influence their perceived thermal comfort. Therefore, this insulation value was included in the systematic approach and calculated from the garment selected by the individual, according to their thermal insulation value.^14^ The level of activity of an individual can also influence their perceived thermal comfort.^28^ However, merely attending a lecture, which is a sedentary activity, would not result in a large differences in the metabolic rate among students, although there may be a difference between the metabolic rate of the students (sitting) and that of the lecturer (standing and/or sitting). The mean score for perceived thermal comfort was derived from the students' thermal sensation and preference score. Following Schweiker et al.,^29^ the three middle votes of the thermal sensation, slightly cool, neutral, and slightly warm, were selected to represent comfortable conditions. Table 1 provides detailed information about how the perceived thermal comfort scale was computed.

**TABLE 1:** Perceived thermal comfort scale

Item	Old value	Original classification	New value	Comfort classification
PTC_sens_	1 2 3 4 5 6 7	Cold Cool Slightly cool Neutral Slightly warm Warm Hot	1 2 4 4 4 2 1	Very uncomfortable Uncomfortable Comfortable Comfortable Comfortable Uncomfortable Very uncomfortable
PTC_pref_	1 2 3 4 5 6 7	Much warmer Warmer A little warmer Neither warmer nor colder A little colder Colder Much colder	1 2 3 4 3 2 1	Very uncomfortable Uncomfortable Slightly uncomfortable Comfortable Slightly uncomfortable Uncomfortable Very uncomfortable

*Note*: PTC_sens_, thermal sensation; PTC_pref_, thermal preference.

#### 2.2.3 Response moderators

Different personal, cultural, climatical, social, and contextual factors can explain differences in individual reactions to the same IEQ.^30^ Therefore, all response moderators were listed after reviewing all of the included studies. Accordingly, age and gender were included in the systematic approach as general response moderators.^6^, ^19^, ^21^ Furthermore, the classroom positions of students and lecturer, the number of students per classroom, and number of students in the classroom were identified as external‐related response moderators.^21^, ^31^

#### 2.2.4 Student responses

In general, people respond physiologically, cognitively, and emotionally to their indoor environments.^11^ To determine how these responses should be measured, all studied physiological, emotional, and cognitive responses were listed. To assess physiological responses to the indoor environment, all examined health symptoms and body‐related issues were listed. A total of 23 health symptoms were identified. Heart rate, blood pressure, melatonin concentration, saliva cortisol concentration, and thirst were identified as body‐related issues. The identified health symptoms were divided into five health categories: (1) dermatological symptoms, (2) tympanic, ophthalmological, and vision‐related symptoms, (3) upper respiratory symptoms, (4) neural behavioral symptoms, and (5) mucosal symptoms.

Table 2 shows all IEQ related health symptoms, their corresponding ICD‐10 health codes of the World Health Organisation,^32^ and related IEQ parameters. These health symptoms were self‐reported.

**TABLE 2:** Self‐reported health symptoms and their relation to the indoor environment

Cat.	Health symptoms (self‐reported)	ICD‐10 code	Relation (Ref.)			
			IAQ	TE	LE	AE
D	Itchy skin, skin irritation, skin rash, dermatological skin problems	R21		○^35^		
E	Itchy eyes, eye irritation, dry eyes, earache, deafness	H57.8; H92.09; H91.90	○^36^			●^34^
R	Dry throat, throat irritation, nasal dryness, nose irritation, sinus congestion, coughing, sneezing, wheezing, respiratory distress	J39	○^37^	●^33^ ○^35^		●^34^
C	Headaches, nausea, lethargy, dizziness	G44; R11; R53; F44‐45	●^34^	●^6^	○^36^	●^34^
M	Mucosal symptoms	R68		○^35^		

*Note*: Cat. = Category; D = Dermatological symptoms; E = Tympanic ophthalmological vision‐related symptoms; R = Respiratory tract (upper respiratory symptoms); C = Central nervous system (neural behavioral symptoms); M = Mucosal symptoms; IAQ = indoor air quality; TE = Thermal environment; LE = lighting environment; AE = Acoustic environment.

○ = Reported relation in study; ● = Reported and confirmed relation based on study outcomes.

To identify possible health symptoms, filter questions were added to the systematic approach for each health category and health issues were specified. To determine whether a reported health symptom is building‐related, a question was added to reveal if the reported symptom (or symptoms) disappeared after leaving the building. If this was the case, the reported health issue may be linked to the IEQ of the building. If not, the reported health issue was excluded from the analysis. In the systematic approach, the number of health issues is reported as perceived physiological health complaints.

The most frequently studied emotional responses, which were also related to students' mental health, were fatigue,^6^, ^38^, ^39^ sleepiness,^22^, ^40^ and tiredness.^34^, ^41^ Four standardized methods for measuring emotional responses were identified from the literature. The first is the Positive And Negative Affect Scales, which focusses on individual resources, activities, and perceptions of the social environment.^42^ The second is the Basic Emotional Process Scale, which assesses the individual emotions in terms of activation, evaluation, orientation, and control.^43^ The third is the Karolinska Sleepiness Scale, which measures perceived sleepiness affecting alertness.^44^ The fourth is the Pittsburgh Sleep Quality Index, which measures the reported sleep quality for one month.^45^ Appendix C presents the structure and items of these emotional response methods.

The classification of the items for assessing students' cognitive responses was complex. Studies have reported students' learning performance while addressing students' cognitive responses. In these cases,^7^, ^8^, ^46^ the framework depicted in Figure 1 facilitated the classification of the identified methods. Following Xiong,^8^ four main categories were identified, that isattention and concentration, memory, perception, and problem‐solving performance. The perceived cognitive response can be measured with items focusing on these four categories. The available information in the studies was used to formulate five items that cover these four categories. Objective cognitive responses can be measured with the use of psychometric tests of neurobehavioral functions. For measuring attention and concentration and memory, respectively, the Go‐No Go task^47^ and the Corsi block test^48^ were selected. The Stroop test was used to measure students' perception, whereas their ability to solve problems was assessed with the Wisconsin card sorting test.^49^, ^50^ These tests were selected as they have been empirically validated and are practically feasible. Feasibility criteria included online availability of the test free of charge and no requirement of special equipment, the ability to perform the test using a mobile phone or laptop, and time‐based efficiency for naturally occurring field experiments (5 min or less).^51^ Table 3 shows the included cognitive response tests.

**TABLE 3:** Cognitive response tests used for the selected categories

Category	Test	Ref.	Link to test
Attention and concentration	Go‐No Go task	47	[link]
Memory	Corsi block task	48	[link]
Perception	Stroop task	49	[link] [link]
Problem‐solving	Wisconsin card sorting test	50	[link]

#### 2.2.5 Academic performance

Students' academic performance is the last category which is affected by IEQ, as depicted in the framework shown in Figure 1. This performance can be divided into students' long‐term and short‐term academic performances. Long‐term performance relates to students' academic performance during an academic semester. Regrettably, no methods were identified for measuring students' long‐term academic performance in the studies. However, we did identify two methods for measuring short‐term performance, which relates to students' academic performance during a lecture. The first was the use of questionnaires to measure the perceived quality of learning, including students' academic performance. Following Lee, Mui, Wong, Chan, Lee, and Cheung,^52^ two items that address students' ability to write (type) and read and an overall statement that addresses students' productivity during the lecture were added to the systematic approach.

The second method entailed administering a content‐related test to measure students' academic performance at the end of the lecture and thus assess knowledge transfer between lecturers and students during class. This test focused on logistical principles and practices covered during the lecture. Students' ability to pay attention during the lecture may affect their ability to remember the content presented during the lecture.^53^ Therefore, our systematic approach followed the procedure of Shelton^54^ and McDonald.^55^ Students would first complete the questionnaire, which evaluated their perceived IEQ, internal responses, and quality of learning. They would subsequently take the academic performance test to measure their ability to recollect the information presented by the lecturer. The above order of implementation increased the time span between the lecture and the content‐related test. Therefore, the students were forced to focus their thoughts first on aspects other than those covered during the lecture. For the pilot study, the test was designed in collaboration with the concerned lecturers and consisted of 10 multiple‐choice questions. The percentage of questions answered correctly reflected students' short‐term academic performance.

#### 2.2.6 Overview of the elements in the composed systematic approach

**FIGURE 5:**
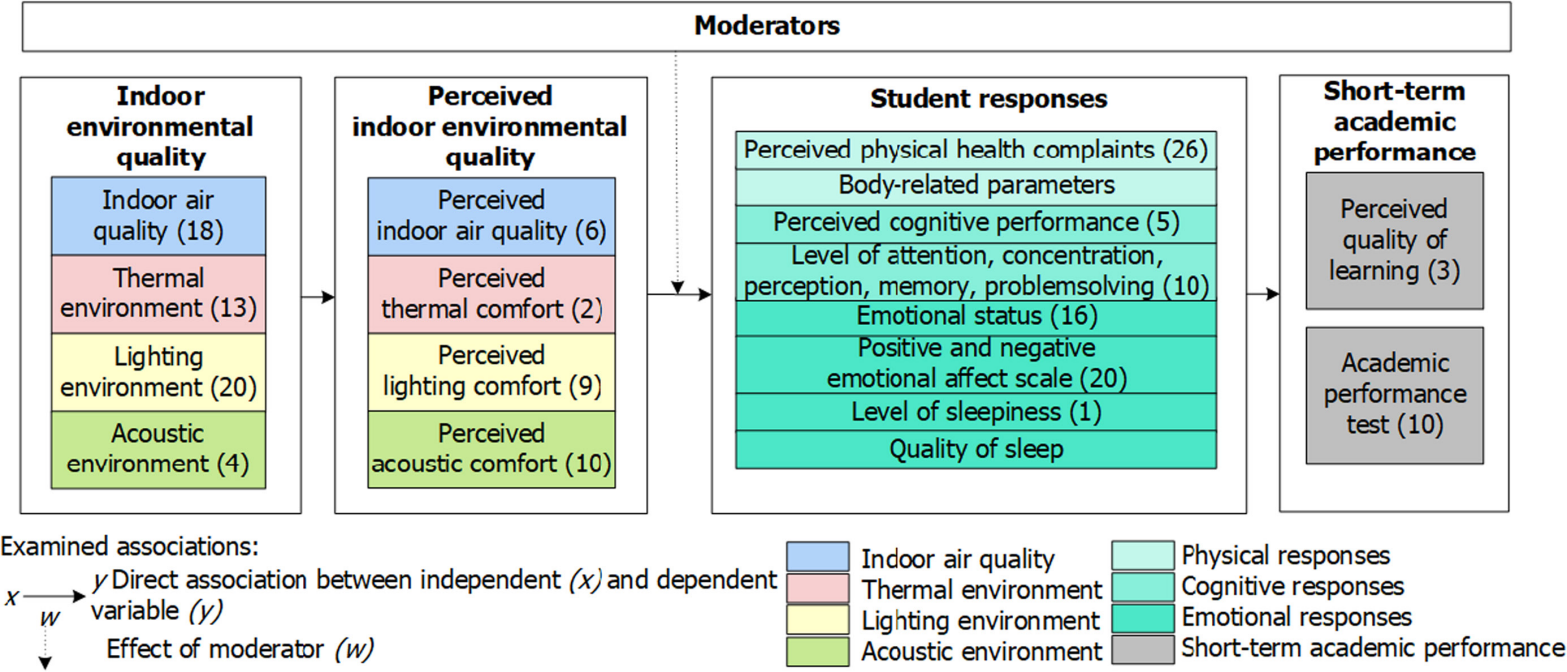
Categories covered in the systematic approach and their mutual relations. The figures in the parentheses indicate the number of items in the questionnaire that cover these categories

The developed systematic approach, which measures the influence of all IEQ parameters on students, addresses four main categories: (1) indoor environment, (2) perceived indoor environment, (3) student responses, and (4) academic performance, as presented in Figure 1. Figure 5 shows how these categories and their mutual relations are covered in the systematic approach, which is based on the information that emerged from the systematic literature review. Because no methods measuring students' long‐term academic performance were identified, the systematic approach only enables assessing the influence of IEQ on students' short‐term academic performance. The Pittsburgh Sleep Quality Index was not included in the approach, because it covers long‐term sleep quality and is, therefore, less applicable. Furthermore, measurements of body‐related parameters, such as blood pressure or saliva cortisol concentration,^21^, ^46^ were not included, as their inclusion would have limited the applicability of the systematic approach. The approach is designed to be applicable in any higher education classroom setting, assuming a steady‐state situation (~20 min acclimation) and lecturer‐student interactions. There are no restrictions regarding the number and size of classrooms or the number of participants. In the next stage, the systematic approach was tested in practice.

## 3 DEPLOYMENT OF SYSTEMATIC APPROACH

### 3.1 Method

A pilot study was conducted to test the systematic approach in February 2020 during the same week in which the first confirmed case of COVID‐19 was reported in the Netherlands. However, the classroom setting and students' attendance were not affected by the pandemic at the time. This pilot study specifically aimed at assessing the applicability and validity of the perception categories relating to thermal comfort, indoor air quality, lighting comfort, acoustic comfort, and cognitive performance, as these categories were modified versions of those referred to in the literature. Existing methods and items, namely emotional response methods and cognitive performance tests, were not tested in the pilot study as these methods were not modified during the development of the systematic approach and their applicability has been demonstrated.^6^, ^8^, ^21^, ^40^

#### 3.1.1 Design set‐up

**FIGURE 6:**
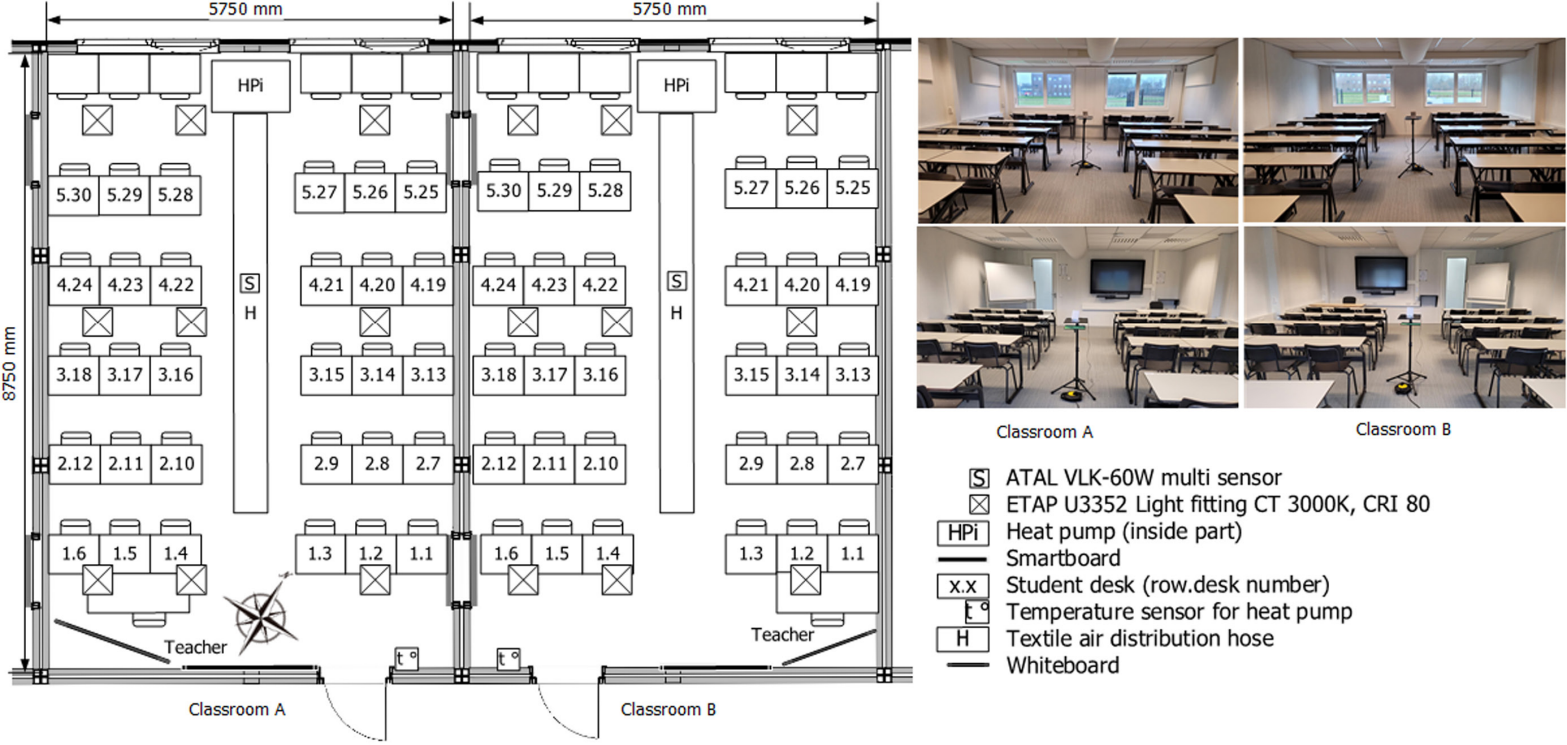
Layout of the classrooms A and B along with four photographs of the A and B interiors (left to right respectively)

In this study, first‐year students of the Hanze UAS School of Business Management, which is in the northern part of the Netherlands, participated as part of their educational programme. These students were selected because they were laypersons who were not versed in building physics. For the pilot study, two heated and naturally ventilated classrooms of the Hanze UAS were selected. These classrooms were equipped with a full air recirculation system to achieve a set air temperature. Outdoor air could enter the classrooms through grilles located above the double glazing. Both classrooms were fitted with nine ETAP U3352 light fittings. Figure 6 presents the floorplan of the classrooms, their orientation, the building facility components in the classroom, and its general visual appearance.

This pilot study was approved by the ethical committee of the Hanze UAS (approval no. 2019.026). Prior to their participation, the students were provided with a general outline of this study and its objective, which was to assess the quality of the classroom. All students who participated in this study signed an informed consent form. As a reward for participation, each student received a voucher for a cup of coffee or tea. The students could end their participation in the study without any consequences at any time. However, none of the students requested to do so or to have their data removed.

#### 3.1.2 Procedure

To determine the indoor air quality, carbon dioxide, particulate matter (PM10 and PM2.5), and volatile organic compounds was measured. To determine the thermal environmental quality, air temperature, and relative humidity were measured. Because of the low thermal mass of the building in which the classrooms are located, the assumption was made that the globe, radiant, and wall temperatures did not differ beyond accuracy specification from the air temperature, which was confirmed in a follow‐up study. The indicators for indoor air quality and thermal environmental quality were measured using an ATAL VLK‐60W multisensor, which was placed in the middle of the classroom at a height of 1.1 meters (see Figure 6).^56^ During the pilot study, this device sent all readings every five minutes to an online platform (www.onlinesensor.nl). These data were used to determine the test conditions. Before the experiment started, air temperature and carbon dioxide concentration were also measured at the front and in the back of the classroom with an ATAL ENV‐MB350NV sensor to determine whether the thermal environmental and indoor air quality in the classroom itself varied. To determine the quality of the lighting environment, the horizontal illuminance level at the desktop of each desk was collected, with the use of a VOLTCRAFT MS‐1300 illuminance measurement device, before the start of the lecture. The students were asked to note their position in the classroom (table number) when they filled in the online questionnaire. The horizontal illuminance level of the desk was linked to the table number. Furthermore, the table number was used to determine the row in which the student sat during the lecture. This row number was used for further analysis as an indicator of the physical distance between the student and the teacher. To determine the quality of the acoustic environment, the background noise and the average reverberation time, at frequencies ranging between 250 and 2000 Hz were measured. Because both classrooms were equipped with the same heat pump and the lecturers involved delivered the same number of lectures, the assumption was made that the ambient sound did not influence students' perceived acoustic comfort. Appendix D presents the measured indicators and details on the accuracy of the measuring equipment.

During the pilot study, four lecturers delivered in total 12 lectures, each lasting approximately 2 h on every weekday apart from Monday. Each lecturer delivered the same number of lectures in classrooms A and B. All participants spent more than 20 min in the classroom; therefore, the assumption was made that all individuals were acclimated to the indoor environment.^19^ After each lecture, the students present were asked to participate in the pilot study. The degree of participation was high, with approximately 90% of all students taking part in the study. There was a short 10‐min break, during which the students stayed in the room, before they filled in the questionnaires. To obtain their IEQ perceptions and responses and to assess their academic performance, all participants first completed the online questionnaire, which measured their perceived comfort, perceived physiological and cognitive responses, and perceived quality of learning. After completing this questionnaire, they took the academic performance test, which comprised 10 questions on topics discussed during the lecture. There was no time limit for the students to complete the questionnaire and test. Those who took the academic performance test received an email the following week with their personal test scores.

#### 3.1.3 Analyses

To determine the validity of the developed systematic approach, all collected data were statistically analyzed. To assess the internal validity of the questionnaire, which addressed the perceived IEQ, cognitive response, and quality of learning, first, the scores on all negative formulated items were reversed. Next, an analysis of Cronbach's alpha (*α*) values was performed to assess the internal consistency of these scales. To determine the normal distribution of the data, Q–Q plots were computed and Shapiro–Wilk tests were performed. Next, the assumed associations (see Appendix E) were tested with linear regressions. The assumed associations of the perceived physical health complaints were analyzed by performing a Poisson regression because this dependent variable consists of “count data.”

The output of the regression analysis was only taken into consideration when it met the following assumptions. The first was the assumption of normality. To determine the normal distribution of the standardized residuals, a probability plot (P–P) plot was computed. When this plot of the residuals appeared to be approximately linear, the assumption of normal distribution was met. When the outcome appeared not to be linear, the distribution of the standardized residuals and unstandardized residuals was analyzed by performing the Shapiro–Wilk test. When the significance level of this test is >.05, the assumption for normality was met. When the regression model did not meet these assumptions, a one‐tailed Spearman test was performed to test an association.

All linear regression models were checked using Cook's diagnostic measure. This value gives a measure of distance per respondent over which the maximum was evaluated. In case of a value that exceeded the cut‐off value 4/n,^57^ the significance of the regression unstandardized coefficients were compared with those from robust regression models.^58^ When this comparison resulted in a different conclusion with respect to the coefficient, it was reported. For multivariate associations the tolerance values should be .10 or higher to rule out multicollinearity.^59^ In the case of multicollinearity, the variable with the lowest bivariate standard correlation coefficient was excluded from the model. In the Poisson regression analysis, the moderation effect was determined by including all moderators separately in the regression model, as covariates or factors. The missing values in all of the linear regression models were excluded listwise. For all tests, the confidence interval (CI) was set at 95%. The lm function (lme4) and the robust lmm function (robustlmm) in R version 3.5.0 (R Foundation for Statistical Computing, 192 Vienna, Austria) and IBM SPSS Statistics Version 28.0.1.0 were used for statistical analyses. To determine the effect of response moderators, a logistic regression path analysis modeling tool was used.^60^

### 3.2 Results

#### 3.2.1 Indoor environment

**FIGURE 7:**
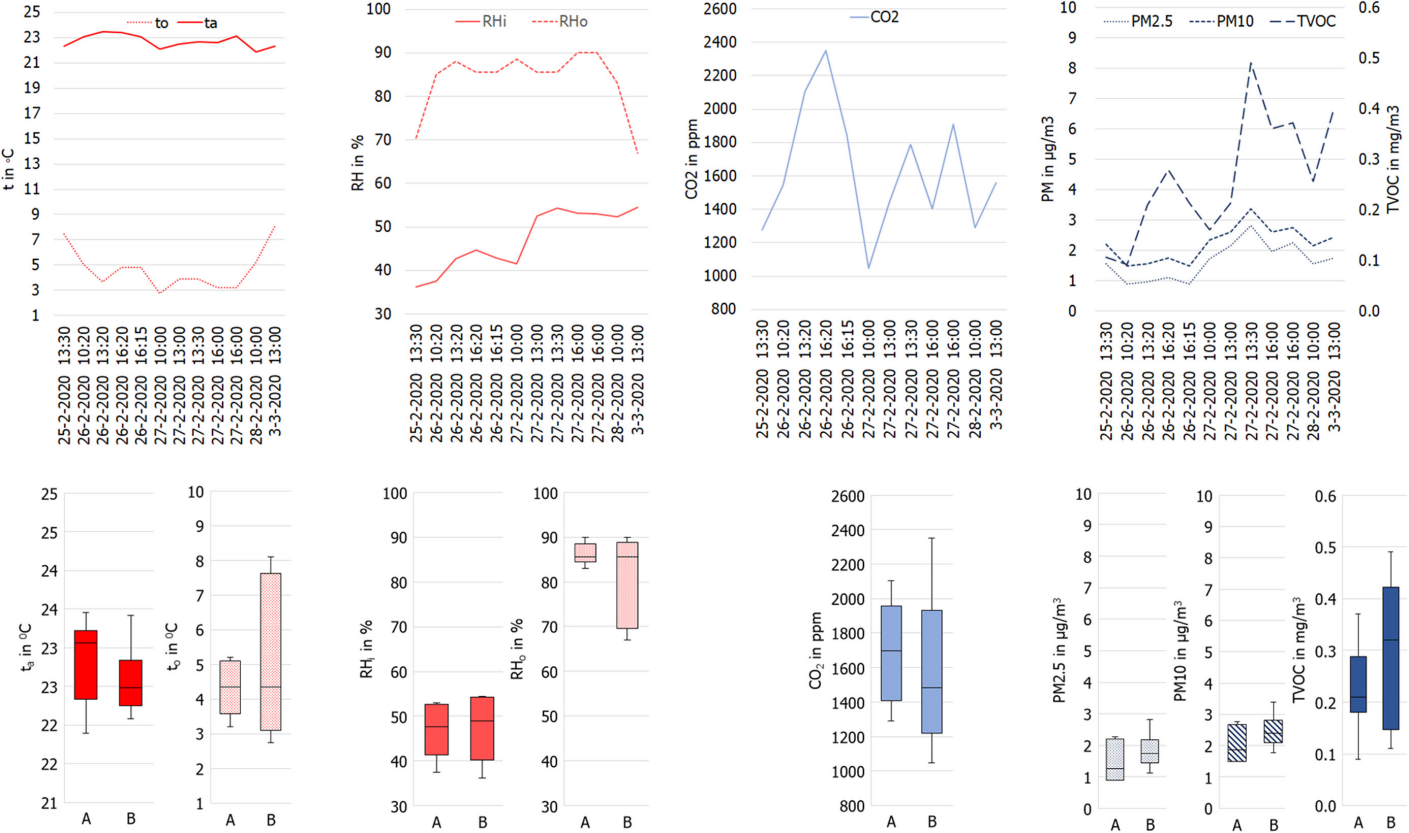
Observed indoor and outdoor thermal environmental conditions (in red color accents) and indoor air quality (in blue color accents) during the 12 observed lectures at the moment students filled in the questionnaire. The line graphs show the natural variations in the classrooms during the experiment; the boxplots show the conditions in classrooms A and B *Note* : RHi = indoor relative humidity; RHo = outdoor relative humidity; ta = air temperature; to = outdoor temperature; CO2 = carbon dioxide; PM10 = particles < 10 µm; PM2.5 = particles < 2.5 µm; TVOC = total volatile organic compounds

The outdoor temperature varied between 3.2 and 8.1°C. The indoor temperature was regulated using the installed heating system and varied slightly, remaining 23°C. The mean air velocity was considered to be <0.09 m/s because the windows in both classrooms were closed during the pilot study; therefore, the assumption was made that this indicator did not influence students' perceived thermal comfort. The average differences in air temperature and carbon dioxide concentration registered in the center of the classroom, compared to those registered at the front and back of the classroom were marginal (+1% and +3% respectively), indicating that the textile air distribution hose of the heating system (see Figure 6) mixed the ambient air sufficiently. The average carbon dioxide concentration outside was approximately 422 ppm.^61^ The amount of daylight in the classrooms was low, because of a window‐to‐floor area ratio of 3% and the North to North‐West orientation of the classrooms that prevented the entry of direct sunlight during the experiment. The measured level of horizontal illuminance of the participants' desktops was 661 ± 162 lux. The major source of sound in the classroom, besides the installed heat pump, was the lecturer's voice. The average reverberation time at frequencies between 250 and 2000 Hz, in classroom A and B were 0.44 and 0.56 s, respectively, and the average background noise in both classrooms varied between 35 and 42 dB(A). Figure 7 shows the natural variations of the thermal environmental and indoor air quality conditions in classrooms A and B.

#### 3.2.2 Perceived indoor environmental quality, response moderators, student responses, and academic performance

Data on the perceived indoor air quality, thermal sensation, thermal preference, acoustic comfort, and lighting comfort were collected. To assess the internal validity, the α‐values were calculated for the perception scales thermal, acoustic, and lighting comfort and indoor air quality. All items contributed to the internal validity of the scales, except for the statement addressing the perceived lighting comfort, namely “In the classroom, the light rarely flickers,” which was therefore excluded. The *α*‐values for the perception scales ranged from 0.70 to 0.89, showing that these scales have considerable reliability.^62^ Average perception scores for these subcategories of perceived IEQ were used for further analyses. The average value for clothing insulation value was 0.5 ± 0.1. With the deletion of the one statement addressing the perceived lighting comfort, the systematic approach for examining the perceived IEQ was adjusted.

Of the five identified response moderators, data of four response moderators were used. The position of the lecturer in the classrooms, as presented Figure 6, was the same during the pilot study and did not vary during the lectures or according to the classrooms used. Therefore, this variable was not analyzed during the pilot study. A total of 163 students, with average age of 19.2 ± 1.6 years participated in the pilot study, of whom 64 were female students. The row average in which the students sat, which reflected the relative distance between the lecturer and the individual student, was row 3 ± 1 and an average of 14 ± 3 students were present during the lectures.

Of the students, 20% reported one symptom, 9% reported two symptoms, 1% reported four symptoms, and 1% reported five symptoms. In addition, students' perceived cognitive response was collected. The α‐value of the perceived cognitive response scale is 0.87, showing that this scale has considerable reliability^62^; therefore, the average perception score of this scale was used for further analyses.

**FIGURE 8:**
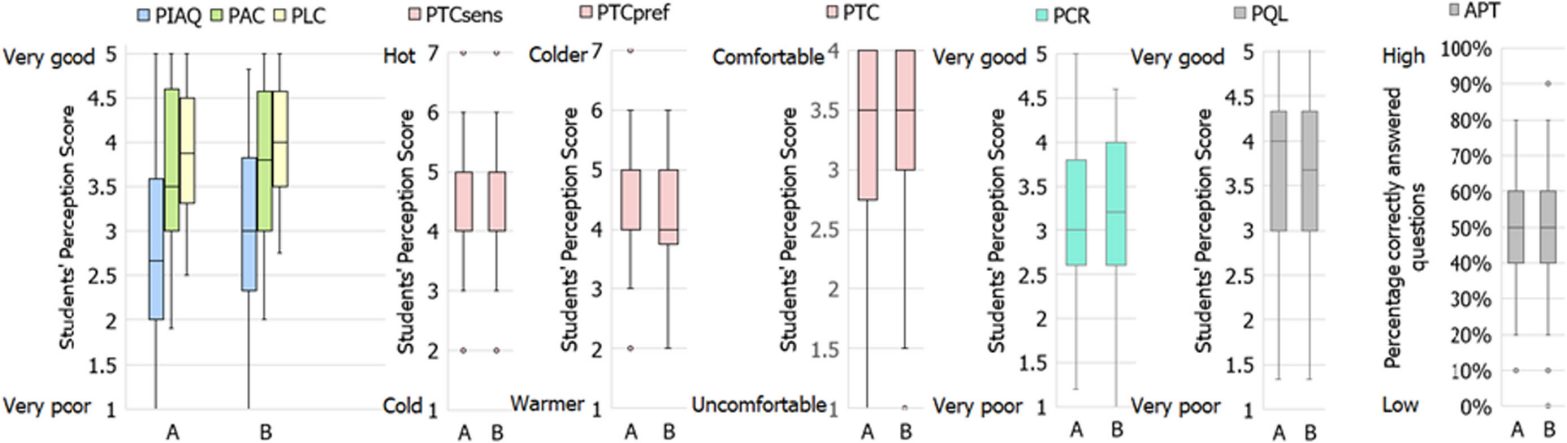
Perceived indoor environmental quality, cognitive response, and quality of learning scores and academic performance test scores *Note* : APT = academic performance test score; PCR = perceived cognitive response; PIAQ = perceived indoor air quality; PLC = perceived lighting comfort; PTCpref = thermal preference; PTCsens = thermal sensation; PTC = perceived thermal comfort

Academic performance was derived from students' perceived quality of learning. The quality of learning items in the questionnaire contributed to the reliability of this perception scale except for the statement “I was very productive during the lecture.” This scale was omitted from further analysis because of the low *α*‐value of 0.68. Furthermore, to measure students' short‐term academic performance, they completed a content‐related test at the end of each lecture. The percentage of questions correctly answered by the students were used for further analyses. Figure 8 presents a summary of the results of the pilot study, with boxplots of all perception and academic performance test scores. Table 4 presents the composition of perception scales, *α*‐values of scales, and the *α*‐value when an item was deleted.

**TABLE 4:** Composition of all perception scales, including α‐values and related items per category and supporting references

Scale	Cat.	Item	Ref.	RS	*α* Del
PTC (*α* = 0.70)	Thermal sensation	Please classify the indoor temperature at this moment: cold, cool, slightly cool, neutral, slightly warm, warm, hot	14, 16, 17, 64, 68	^a^	—
	Thermal preference	At this moment, would you prefer to feel much warmer, warmer, a little warmer, neither warmer nor colder neutral a little colder, colder, much colder	16, 17	^a^	—
PIAQ (*α* = 0.82)	Quality of air	There is some stale air in here	12,15, 25	✓	0.76
		There is a lot of fresh air in here	22, 23		0.80
	Ventilation	The classroom is properly ventilated	15		0.79
	Odor character and intensity	There is a bad smell in here	12, 22, 25, 37	✓	0.78
	Moisture	The air is dry in here	24, 25	✓	0.80
		The air is dusty in here	25	✓	0.80
PLC (*α* = 0.77)^2^	Amount of (day)light	The visual comfort in the classroom is very bad	13	✓	0.73
		I can see well in this light	25, 72, 73		0.73
		The illumination provided by projectors appears to be inadequate	13, 25, 72	✓	0.70
		It is dark in the classroom	25	✓	0.73
	Flickering	In the classroom the light rarely flickers^1^	21, 25		0.77
	Reflections and glare	In the classroom, I frequently experience annoying reflections produced from the outside	13, 21, 25, 73	✓	0.68

	Color sensation	In the classroom, I frequently experience unpleasant color sensations	13, 25	✓	0.71
	Contrast	In the classroom, windows create dark areas	13, 25	✓	0.71
PAC (*α* = 0.89)	Noise from within the classroom	Students moving and mingling in the classroom interfere with my ability to hear in the classroom	13, 25, 31, 34, 71	✓	0.88
		Noise from the instrumentation used in the classroom interfere with my ability to hear in the classroom	13, 25, 31, 34, 71	✓	0.89
	Noise from outside the classroom	Students speaking outside the classroom interfere with my ability to hear in the classroom	25, 34	✓	0.89

		Noise from people or instrumentation outside the classroom but inside the building interfere with my ability to hear in the classroom	13, 25, 71	✓	0.88

	Noise disturbance	I experience prolonged noise disturbance	13	✓	0.89
		I experience short noise disturbance	13	✓	0.88
		Noises that occur only once interfere with my ability to hear in the classroom	13	✓	0.88
		Noises that occur occasionally interferes with my ability to hear in the classroom	13,	✓	0.88
		The noises I hear in the classroom bother me	31, 71	✓	0.88
		The noise disturbs my concentration	13	✓	0.88
PCR (*α* = 0.87)	Alertness	I was very alert during the lecture	22		0.84
	Concentration	I was able to concentrate well during the lecture	13, 38, 39, 78		0.84
	Memory	I can remember the content of the lecture well	20		0.83
	Perception	I was able to understand the lecture well	8, 52		0.84
	Problem‐solving	I was able to solve complicated problems during lecture well	8		0.84
PQL (*α* = 0.68)	Productivity	I was very productive during the lecture	36		0.79
	Reading	I was able to read well during the lecture	52		0.42
	Typing	I was able to type well during the lecture	52		0.47

*Note*: APT = academic performance test score; PCR = perceived cognitive response; PIAQ = perceived indoor air quality; PLC = perceived lighting comfort; PTC = perceived thermal comfort; PQL = perceived quality of learning.

Cat. = perceived indoor environmental quality category; RS = reverse score was used to compute scale; α del. = α‐value when item is deleted.

^a^ Table 1.

^1^ *α*‐value after removing items from scale.

^2^ Item was deleted before calculating mean score.

### 3.3 Data Analyses

Multiple regression analyses were conducted to examine how well the independent variables (*x*) could predict the dependent variable (*y*). First, the assumption that the IEQ influences the perceived IEQ was tested. The outdoor conditions reflected in the outdoor temperature and relative humidity were excluded from the data analyses because the students spent more than 20 min in the classroom before they evaluated the thermal environment. Consequently, it was presumed that this time would be sufficient for their bodies to acclimatize to these circumstances.^19^ The multiple regression model of the indoor air quality and the perceived indoor air quality showed multicollinearity between PM10 and PM2.5. PM10 had the lowest bivariate standard correlation coefficient and was therefore excluded for further analyses. An analysis of the lighting environment revealed that only the horizontal illuminance varied during the pilot study. Therefore, this indicator was included in the analyses. Furthermore, the reverberation time was included in the analysis as an acoustic indicator, because the reverberation time of classroom A, compared to that of classroom B, was the only indicator that differed between the classrooms during the pilot study. A bivariate regression analysis of the assumed relation between students' clothing insulation value, as the independent variable, and students' perceived thermal comfort, as dependent variable, did not reveal a significant relation. Therefore, this relation was not further analyzed. Although the perceived IEQ scales did not pass the Shapiro–Wilk test, the Q–Q plots did not reveal large deviations from normality.

Next, the assumption that the perceived IEQ influences students' responses was tested. Although students' self‐reported physiological health complaints and their perceived cognitive performance did not pass the Shapiro–Wilk test, Q–Q plots did not reveal large deviations from normality. The Q–Q plot of the perceived physiological health complaints revealed a skewed distribution of data, indicating that this variable was not normally distributed.^19^ The robust models showed higher estimates of all variables except for the model for perceived cognitive performance. However, this estimate was not significant and therefore did not lead to a different conclusion regarding this coefficient. Table 5 presents the outcome of all linear regression analyses or Spearman's rho, when the assumptions for regression were not met.

**TABLE 5:** Outcome of bivariate and multivariate linear regression analyses and Spearman rho coefficient for the data collected

*x*	*y*	*A*	*β*	*R* ^2^_adj_	*F*‐value	Model sig.	df_regr_	df_res_	SP_coef_
CO_2_ PM2.5 TVOC	PIAQ^m^	Yes	−0.143 −0.202* −0.178*	0.107	7.437	***	3	158	
RH_i_ t_a_	PTC_sens_^m^	Yes	0.239 0.112	0.042	4.571	*	2	160	
RH_i_ t_a_	PTC_pref_^m^	Yes	0.355*** 0.104	0.104	10.389	***	2	160	
E_hor_	PLC^b^	No^1^	−0.024	−0.006	0.088		1	158	−.039
RT	PAC^b^	No^1^	−0.023	−0.006	0.084		1	159	−.002
PIAQ PTC PLC PAC	PPHC^P^	Yes	−0.7662^2^ 0.037^2^ −0.010^2^ −0.301^2^	0.465*** ^3^ 1.038^3^ 0.990^3^ 0.740* ^3^	n/a	***	4	155	
PIAQ PTC PLC PAC	PCR^m^	Yes	0.080 0.060 0.152 0.045^4^	0.033	2.369	*	4	155	
PCR	APT^m^	Yes	0.269***	0.060	6.055	***	2	155	
PPHC			−0.004						

*Note*: APT = academic performance test score; PCR = perceived cognitive response; PIAQ = perceived indoor air quality; PLC = perceived lighting comfort; PPHC = perceived physiological health complaints; PTC_pref_ = thermal preference; PTC_sens_ = thermal sensation; PTC = perceived thermal comfort.

*x* = independent variable; *y* = dependent variable; A = assumptions met; *β* = standardized coefficient beta; *R* ^2^_adj_ = squared regression coefficient; df_reg_ = degrees of freedom of regression; df_res_ = degrees of freedom of residual; SP_coef_ = Spearman's rho correlation coefficient; ^b^ = bivariate linear regression analyses; ^m^ = multivariate linear regression analyses; p = Poisson regression analyses.

* Correlation is significant at the 0.05 level (one‐tailed).

** Correlation is significant at the 0.01 level (one‐tailed).

*** Correlation is significant at the 0.001 level (one‐tailed).

^1^ Probability plot of standardized residuals and unstandardized residuals revealed a non‐linear relationship and the Shapiro–Wilk test revealed a significance level of *p* < 0.05. Therefore, the assumption for normality was not met.

^2^ Coefficient estimate.

^3^ Exponentiated value of coefficient.

^4^ Robust estimate value was lower.

**FIGURE 9:**
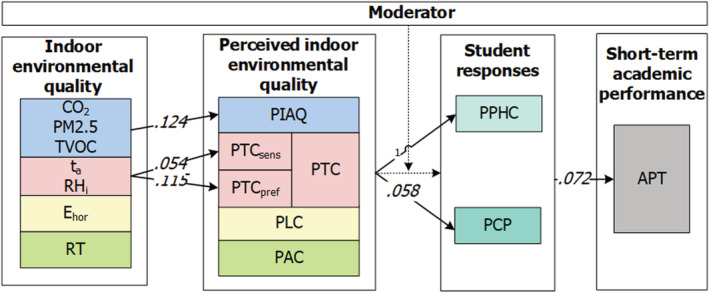
Significant multivariate linear regression *R* ^2^ values between independent and dependent variables. See footnote to Figure 5 for explanation of symbols and shading *Note* : APT = academic performance test score; PCR = perceived cognitive response; PIAQ = perceived indoor air quality; PLC = perceived lighting comfort; PPHC = perceived physiological health complaints; PTC_pref_ = thermal preference; PTC_sens_ = thermal sensation; PTC = perceived thermal comfort; 1 = only exponentiated values of coefficient could be calculated, see Table 5

Furthermore, the interactions were analyzed of the response moderators (*w*), namely age, gender, classroom position of students, and number of students present in the classroom during lecture, with the independent (*x*) and the dependent variables (*y*). The aim was to determine whether the effect of *x* on *y* was moderated by *w*; that is, whether the size or sign of the effect of *x* on *y* varied with *w*. However, no significant moderation effects were found. Figure 9 depicts significant multivariate linear regression *R* ^2^ values between independent and dependent variables.

## 4 DISCUSSION

The main objective of this study was to develop and validate a systematic approach that would enable an assessment of the combined influence of IEQ parameters on students' perceptions, responses, and academic performance. To develop this systematic approach, methods derived from 54 included publications were reviewed. This paragraph specifies those methods used to measure the influence of IEQ on students and their short‐term academic performance. Possible adjustment behaviors in result to any discomfort experienced, such as opening windows or taking off or putting on clothes, were not included because the composed approach focuses on the consequences of experienced comfort or discomfort *ceteris paribus*, as induced by the IEQ. However, future studies that specifically focus on students' comfort level should account for the possibilities the students have to adjust indoor environmental circumstances. How the IEQ affects students and their performance, in this case with reference to multisensory influences on human responses, was ascertained from previously published work.^4^, ^74^

Although the identified publications did not always provide detailed information about the applied method, all methods could be linked to a category of the framework (see Figure 1), which demonstrates the practical applicability of this framework. The identified methods provide a rich and diverse perspective of how the influence of indoor environmental parameters on students' perceptions, responses, and short‐term academic performance can most effectively be measured. The designed systematic approach combines these methods, enabling researchers to study both the individual and the combined influence of all indoor environmental parameters on students. This holistic character of the systematic approach responds to the need to develop human response models to assess the influence of multiple environmental parameters on performance.^10^ Students' emotional response and cognitive performance were not tested during the pilot study. The current systematic approach measures the immediate interaction and short‐term academic performance of the students, and thus does not measure the long‐term effects of the IEQ on students. However, application of this protocol over a longer period and inclusion of long‐term academic performance measures, for example, students' grades, may reveal long‐term effects. Below, the development, the applicability, and the testing of the systematic approach, is discussed.

### 4.1 Development of the systematic approach

The developed systematic approach, which measures the combined influence of all IEQ parameters on students, addresses four main categories: (1) indoor environment, (2) perceived indoor environment, (3) student responses, and (4) academic performance. To determine the IEQ, 54 indicators were identified that provide detailed information about the actual IEQ. In future studies, relevant indicators should be selected from this list of 54 indicators to measure the IEQ, depending on the aim and scope of the study. When studying the influence of the IEQ on students, inclusion of the Pittsburgh Sleep Quality Index and body‐related parameters can be considered, depending on the study's aim. Furthermore, body composition and sweat excretion can considered when assessing students' thermal comfort, which may help to explain the variations in thermal comfort under similar conditions.^75^, ^76^

A comprehensive questionnaire was developed by including the methods that address the perceived IEQ, cognitive responses, and quality of learning. The validity of this questionnaire was tested and confirmed in the pilot study. To cover the cognitive response categories (attention, perception, memory, and problem‐solving),^8^ empirically validated and practically feasible tests were selected. However, the tests which were identified during the initial search, were not included in the pilot study, as they were already validated in earlier studies. The advantage of using these existing tests^6^, ^7^, ^8^, ^46^, ^54^ is that a comparison with the results of these earlier studies becomes possible. However, possible disadvantages are that not all tests can be easily applied in practical settings and that it sometimes takes more time to determine the students' individual scores. The practical applicability of the selected test for the systematic approach will be tested in a follow‐up study.

### 4.2 Applicability of systematic approach

The developed systematic approach was deployed in a real‐life setting to assess the applicability and validity of new perception scales and to test academic performance, which could not be determined from the information available in the included studies. The internal consistency of the systematic approach measuring the perceived IEQ is acceptable for all scales (*α* > 0.70). However, this evaluation of the internal consistency led to the exclusion of one item, *in the classroom*, *the light rarely flickers*. The negative contribution of this item to the perceived lighting comfort scale can be explained by the fact that both classrooms were equipped with high‐quality LED armatures. In another real‐life setting, with lower quality lighting fittings, assessment of this item may be necessary.

None of the identified studies assessed the general health of respondents. This topic, therefore, was not addressed in the pilot study. In general, a dysfunction, such as deafness, color blindness, or sickness, could influence individuals' response to IEQ and their performance, and this should be considered when analyzing results. An additional question, which assesses this topic, could be added to the systematic approach to incorporate awareness of this fact. The moderation effect of the number of sleeping hours, sleep quality, and room temperature at home were not included in the original systematic approach. However, these variables may moderate students' responses and academic performance and may be added to the systematic approach in future studies.^6^, ^40^ Completing the developed questionnaire, covering the perceived IEQ, physiological and cognitive response, and the quality of learning takes approximately 10 minutes and requires the availability of a mobile phone, laptop or desktop computer.

### 4.3 Testing systematic approach

The pilot study aimed at assessing the applicability and the validity of the systematic approach for simultaneously assessing IEQ parameters in classrooms and focused specifically on those categories that were altered during the development of the approach. During this study, the indoor environment of two classrooms was not actively manipulated, resulting in similar conditions in both classrooms and limited natural variations. However, these natural variations confirmed, to some extent, the assumed associations, as presented by Bitner.^11^ Here, significant associations or the absence of assumed associations are discussed. However, the pilot study was not intended to collect evidence about the influence of the IEQ on students. GPOWER was used to determine the statistical power of the collected data.^77^ The achieved power (1−*β*) for a bivariate normal model (one‐tailed) is sufficient (>0.80) to evaluate the assumed associations, given a relatively small expected effect of 0.20, an *α* of 0.05, and a sample size of 163.

Of the indicators measured in the pilot study, the indicators for the indoor air quality and indoor humidity showed significant associations (*p* < 0.05) with their related perception, revealing the construct validity of these indicators. Furthermore, significant associations (*p* < 0.05) between the perceived IEQ scales and the perceived physiological health complaints as well as the perceived cognitive response were observed, confirming the construct validity of these variables. Finally, a significant association (*p* < 0.001) was observed between the perceived cognitive response and short‐term academic performance. The students reported their perceived cognitive response before they started the academic performance test. However, the explained variance of this perceived cognitive performance on actual academic performance was limited.

The limited variations of the actual IEQ may explain why the observed bivariate and multivariate standardized coefficients are relatively small. Furthermore, the multivariate linear regression model of the actual and perceived indoor air quality showed that indicators for particulate matter (PM10, PM2.5) caused multicollinearity; indicating that one indicator for determining the influence of particulate matter (PM2.5) may be sufficient. The observed indoor air temperature (23.0 ± 0.4°C), as an indicator for the thermal environment, was not associated with the related perception scales for thermal comfort, possibly because of limited temperature variations during the study. The insulation value of the clothing was not associated with the indicators for the thermal environment or with the thermal perception scales, thus confirming the findings of Mishra et al.^19^ However, it might be relevant to assess students' ability to adjust their clothing when they experience thermal discomfort, and this item could be added to the systematic approach.^19^ Furthermore, the assumption was made that the students were fully acclimatized at the time they evaluated their thermal sensation and preference.^19^ Therefore, the effect of the outside conditions during the pilot study, representing winter conditions in the Netherlands, on students' thermal comfort was not further analyzed. However, climatic and seasonal differences may affect students' adaptive processes and therefore could be added to the protocol, when applicable.^29^

The horizontal illuminance was the only studied indicator for the lighting environment that varied during the pilot study. However, this indicator was not correlated with the related perception scale. The reason may be that the average horizontal illuminance was relatively high (between 514 and 715 lx), low levels of horizontal illuminance levels (<300 lx) were not observed. There was a small difference between classrooms in terms of the reverberation time (0.12 s), which was the only indicator considered for the acoustic environment that varied during the pilot study. However, it did not influence students' perceived acoustic comfort in the classrooms.

The *α*‐values were acceptable for all indoor environmental perception scales.^62^ These perception scales were calculated from the students' individual scores on at least five related statements, except for students' perceived thermal comfort which was derived from students' thermal sensation and preference scores. This assumed relation, see also Table 1, is only valid for an average of a large group, but does not hold necessarily for individual votes.^29^ The questionnaire covering the scale for perceived lighting comfort only addresses general visual aspects in the classroom. Desk‐level lighting conditions were not addressed. These issues could explain the absence of an association between the horizontal illuminance and the related perception scale. Therefore, items that address the perceived task lighting conditions may improve the content validity in the systematic approach. Furthermore, items on perceived acoustic comfort only addressed the perceived noise from within and outside the classroom along with noise disturbance. The ability to hear the lecturer's voice, which entails speech intelligibility and is influenced by the reverberation time in a classroom, was not addressed. Items that address speech intelligibility may also improve the content validity of the systematic approach.

## 5 CONCLUSIONS

The developed systematic approach allows researchers to examine the combined influence of multiple environmental parameters on students' perceptions, responses, and short‐term academic performance. As a result, this approach contributes to the need to develop human response models, which enable the influence of multiple environmental parameters on performance to be assessed and which account for the differences between individuals and their responses to the actual and perceived IEQ.

In a pilot study, associations were observed between the actual IEQ indicators, perceived IEQ, students' responses, and students' short‐term academic performance, confirming the ecological validity of this approach. Significant associations (*p* < 0.05) between IEQ indicators, students' perceptions of the indoor environment and their reported physiological and cognitive responses were derived. Finally, students' short‐term academic performance was found to be significantly associated with their perceived cognitive performance (*p* < 0.01). These observed associations confirm the construct validity of the systematic approach for these categories. However, not all assumed associations were confirmed in the pilot study. The validity of the systematic approach to investigate the influence of lighting and the acoustic environment has yet to be determined.

Application of the composed systematic approach facilitates future measurements of the influence of individual or combined IEQ parameters on students' short‐term academic performance. Moreover, future studies could also examine the influence of long‐term exposure to certain IEQ conditions and their impact on students' long‐term academic performance. To make this type of research possible, the current systematic approach should be supplemented with an approach to measure long‐term exposure and its influence on students' long‐term academic performance. A potential option could be to look at students' grades, for example, before and after a renovation that has improved the IEQ.

## FUNDING INFORMATION

This work was supported by the Department of Facility Management and the Executive Board of Hanze University of Applied Sciences, Groningen, the Netherlands.

## CONFLICT OF INTEREST

The authors declare that they have no known competing financial interests or personal relationships that could have influenced the work reported in this paper.

## Supporting information


Appendix

## Data Availability

The data that support the findings of this study are available on request from the corresponding author. The data are not publicly available due to privacy or ethical restrictions.
