# Supplementary material for: A systematic approach to quantify the influence of indoor environmental parameters on students' perceptions, responses, and short‐term academic performance
Source: Indoor Air. 2022 Oct 17;32(10):e13116. doi: 10.1111/ina.13116 (PMC9828016; doi:10.1111/ina.13116)
Supplement: Supplementary file 1 — Appendix [file INA-32-0-s001.docx]

**Appendix A** Nomenclature

| *Indoor air quality (IAQ)*  AC allergen concentration [units/mL]  AER air exchange rate [ac/h]  Cl_2_ chlorine [ppm]  CO carbon monoxide [mg/m³]  CO_2_ carbon dioxide [ppm]  CO_2o_ carbon dioxide outside [ppm]  D dust [g]  HCHO formaldehyde [µg/m^3^]  NO_2_ nitrogen dioxide [µg/m³]  O_3_ ozone [µg/m³]  PM10 particles <10 µm [µg/m³]  PM2.5 particles <2.5 µm [µg/m³]  PSV personal supply ventilation  SD settled dust [g/g_dust_]  SO_2_ sulphur dioxide[µg/m³]  TSP total respirable suspended particulate matter  TVOC total volatile organic compounds [mg/m³]  VB viable bacteria [cfu/m^3^]  VM viable mouts [cfu/m^3^]    *Thermal environment (TE)*  CLO clothing insulation value  RH_i_ indoor relative humidity [%]  RH_o_ outdoor relative humidity [%]  t_a_ air temperature [◦C]  t_f_ floor temperature [◦C]  t_g_ globe temperature [◦C]  t_o_ outdoor temperature [◦C]  t_op_ operative temperature [◦C]  t_r_ radiant temperature [◦C]  t_r_asym_ radiant asymmetry  temperature [µm]  t_w_ temperature of walls [◦C]  t_wb_ wet-bulb temperature [◦C]  v_a_ air velocity [m/s].  *Acoustic environment (AE)*  L_Aeq_ background noise or ambient noise [dB(A)]  F_0_ fundamental frequency [Hz]  RT reverberation time [sec]  SPL sound pressure level [dB]  SPL_a_ A-weighted sound pressure level [dB(A)]  SPL**_c_** C-weighted sound pressure level [dB(C)]  *Lighting environment (LE)*  Cc contrast  CCT correlated colour temperature [K] | CRI colour rendering index  CT colour temperature [K]  DF daylight factor [%]  DFC daylight factor contour  E_amb_ ambient illuminance (illuminance) [lux]  E_c_ cylindrical illuminance [lux]  E_hor_ horizontal illuminance[lux]  E_ver_ vertical illuminance [lux]  L_c_ average ceiling luminance [cd/m^2^]  Lf luminous flux [lm/W]  L_og_Ch chloropic lux [lux]  L_og_Cy cyanopic lux [lux]  L_og_Er erythropic lux [lux]  L_og_Ph photopic lux [lux]  L_og_Rh rhodopic lux [lux]  L_w_ average wall luminance [cd/m^2^]  SPD spectral power distribution  U_0_ illuminance uniformity  *Abbreviations*  AP academic performance  APT_(adj)_ (adjusted) academic performance test score  BEPS basic emotional process scale  BEPSA BEPS activation  BEPSC BEPS control  BEPSE BEPS evaluation  BEPSS BEPS emotional status  BEPSO BEPS orientation  CBS Corsi block score  CR cognitive response  DSS digit span score  ER emotional response  F_rt_ Flanker reaction time [sec]  IR internal response  KSS Karolinska sleepiness scale  NAS negative affect scale  PAC perceived acoustic comfort  PAS positive affect scale  PCR perceived cognitive response  PIAQ perceived indoor air quality  PIEQ perceived indoor environmental quality  PLC perceived lighting comfort  PPHC perceived physiological health complaints  PQL perceived quality of learning  PR physiological response  PTC perceived thermal comfort  PTC_pref_ thermal preference  PTC_sens_ thermal sensation  STR Stroop test  WCS Wisconsin card sorting test  w_ext_ external-related moderators  w_gen_ general moderators |
| --- | --- |
|  |  |

**Appendix B** Summary of included studies for the systematic approach

| Reference | A | n | C | TE | LE | AE | IAQ | CAT | Studied variable | Applied method |
| --- | --- | --- | --- | --- | --- | --- | --- | --- | --- | --- |
| Afren et al. | ✓ | n/a | n/a | RH_i_, RH_o_, t_a_, t_o_, v_a_ |  |  |  | PTC | Thermal comfort | Self-reported thermal preference on the ASHRAE-55 on a 3-points scale (very hot, hot, adequate) |
| Almeida et al. | ✓ | 89^1^ 96^1^ | 2 | CLO, RH_i_, t_a_, t_f_, t_r_, t_r_asym_, v_a_ |  |  |  | PTC | Thermal comfort | Self-reported thermal sensation (Fanger 7-points scale, ranging from -3 to +3, corresponding to very cold and very hot and 0 being the thermal neutral condition) |
| Ashrafi and Naeini | ✓ | 30 | 1 | t_a_ | E_amb_ | L_Aeq_ |  | PIAQ | Ventilation, breathing air | Self-reported suitability |
|  |  |  |  |  |  |  |  | PLC | Lighting | Self-reported suitability |
|  |  |  |  |  |  |  |  | PAC | Noise | Self-reported suitability |
|  |  |  |  |  |  |  |  | CR | Subjective concentration | Self-reported inability in concentration |
|  |  |  |  |  |  |  |  | PR | Health | Self-reported level of environmental health |
| Attia et al. | ✓ | 265 | 1 |  |  |  |  | PTC | Thermal comfort | Self-reported temperature (5-point rating scale too hot/ cold) |
|  |  |  |  |  |  |  |  | CR | Distractions in classroom | Self-reported level of distractions |
| Bajc et al. | ✓ | 40 | 1 | RH_i_, t_a_, t_o_, t_r_, v_a_ |  |  | CO_2_, CO_2o_ | PTC | Overall and local thermal comfort | Self-report sensation |
|  |  |  |  |  |  |  |  | CR | Remembering information | Non-academic texts were read to subjects; after the reading, subjects were asked to answer five questions from the text that had been read |
| Bidassey-Manilal et al. | ✓ | 252 | 5^2^ | t_a_, v_a_ |  |  |  | ER | Tiredness | Hourly symptom log per day |
|  |  |  |  |  |  |  |  | PR | Respiratory tract (upper respiratory symptoms) | Self-reported ability to breath |
|  |  |  |  |  |  |  |  | PR | Central nervous system (Neural behavioural symptoms) | Self-reported headaches, nausea, lethargy, dizziness |
|  |  |  |  |  |  |  |  | PR | Health | Self-reported level of dehydration (thirsty) |
| Castilla et al. | ✓ | 854 | 1 |  |  |  |  | CR | Reading, writing, reflecting, discussing, paying attention | Self-report ability |

| Reference | A | n | C | TE | LE | AE | IAQ | CAT | Studied variable | Applied method |
| --- | --- | --- | --- | --- | --- | --- | --- | --- | --- | --- |
| Castilla et al. | ✓ | 918 | 1 |  |  |  |  | PIAQ | Indoor air quality | Self-reported level of ventilation and damp air |
|  |  |  |  |  |  |  |  | PAC | Acoustic comfort | Self-reported level of silence |
|  |  |  |  |  |  |  |  | PTC | Thermal comfort | Self-reported level of "good temperature" and comfort |
|  |  |  |  |  |  |  |  | PLC | Daylight, artificial lighting, well lit | Self-reported level |
|  |  |  |  |  |  |  |  | CR | Subjective concentration | Self-reported level |
| Castilla et al. | ✓ | 427 | 1 |  |  |  |  | PLC | Visual comfort | Self-reported level of attractiveness, stimulation, and cosiness (attractive, good daylight, stimulating, comfortable, warm, cosy, pleasant, natural, dim (subtle), well lit, good artificial lighting, efficient, cutting edge technology, cosy, surprising, amazing, original, interesting, stimulating, suggestive, eﬃcient, uniform, homogeneous, balanced, orderly, cheerful, colourful, friendly, lively, dynamic, beautiful, enabling, glaring (dazzling), intense, brilliant, calm, quiet, soft, clear, sharp (deﬁned), with quality (rich), bright, functional, convenient, comfortable) |
| Corgnati et al. | ✓ | 427 | 1 | RH_i_, t_a_, t_r_, v_a_ |  |  |  | PTC | Thermal comfort | Self-reported acceptability (at this moment, do you consider the thermal environment acceptable or not?), thermal preference (at this moment, would you prefer to feel warmer, cooler or no change), thermal sensation (Fanger 7-points scale, ranging from -3 to +3, corresponding to very cold and very hot and 0 being the thermal neutral condition) |
| Castro-Martínez et al. | ✓ | 141 24 | 1 |  |  | RT |  | CR | Level of attention | Average of the times students look away to a specific point. This average is calculated from the measurement and evaluation of the images of each participant’s |
|  |  |  |  |  |  |  |  | AP | Recognition | Questionnaire with words belonging and not belonging to the lecture, but related to the subject |
| Chin and Saju | ✓ | 80 | 1 |  |  | L_Aeq_, SPL |  | PAC | Annoyance | Social and socio-acoustic surveys (noise sources which affect students) |
| Choi et al. | ✓ | 15 | 2 | RH_i_, t_a_ | L_og_Ch, L_og_Cy, L_og_Er, L_og_Ph, L_og_Rh, E_amb_, CT | L_Aeq_ |  | PLC | Visual comfort | Self-assessment on a 100 mm visual analogue scale |
|  |  |  |  |  |  |  |  | ER | Morning drowsiness, relaxation | Self-assessment on a 100 mm visual analogue scale |
|  |  |  |  |  |  |  |  | ER | Subjective sleepiness | Karolinska Sleepiness Scale, a nine-point scale ranging from 1 (extremely alert) to 9 (extremely sleepy, fighting sleep) |
|  |  |  |  |  |  |  |  | ER | Sleep quality | Pittsburgh Sleep Quality Index |
|  |  |  |  |  |  |  |  | PR | Health | Level of salvia cortisol concentration and melatonin concentration |

| Reference | A | n | C | TE | LE | AE | IAQ | CAT | Studied variable | Applied method |
| --- | --- | --- | --- | --- | --- | --- | --- | --- | --- | --- |
| Chowdhury et al. | ✓ | 480 | 1 |  |  | SPL_ac_ |  | PAC | Acoustic comfort | Self-reported level of perceived traffic induced noise, noises from corridors and other rooms, and noise generated by themselves |
|  |  |  |  |  |  |  |  | CR | Subjective concentration | Self-reported impaired concentration in their job |
|  |  |  |  |  |  |  |  | PR | Ear | Self-reported earache and deafness |
|  |  |  |  |  |  |  |  | ER | Tiredness | Self-reported tiredness |
|  |  |  |  |  |  |  |  | PR | Respiratory tract (upper respiratory symptoms) | Self-reported respiratory distress |
|  |  |  |  |  |  |  |  | PR | Central nervous system (Neural behavioural symptoms) | Self-reported headaches |
| Ellis | n/a | n/a | n/a |  |  |  |  | PAC | Reverberation time | Application of flooring materials |
| End et al. | ✓ | 71 | 2 |  |  | L_Aeq_ |  | AP | Transfer of academic information | Academic information was presented through a video; the transferred knowledge was tested with two multiple-choice questions that assessed students' ability to recognize factual video content. |
| De Abreu-Harbich et al. | ✓ | 200 | 1 | RH_i_, RH_o_, t_a_, t_o_, v_a_ |  |  |  | PTC | Thermal comfort | Self-reported comfort (do you think this environment is? (comfortable, a little comfortable, uncomfortable, very uncomfortable, extremely uncomfortable), tolerance (this environment, in your opinion, regarding temperature, is it? (perfectly tolerable, a little difficult to tolerate, difficult to tolerate, very difficult to tolerate, intolerable), acceptance (taking into account only your personal preference, do you accept or reject the thermal conditions of this environment? (accept, reject), acceptance (at this moment do you prefer this environment? (much warmer, warmer, a little warmer, neither warmer nor colder (neutral), a little colder, colder, much colder), thermal sensation (Fanger 7-points scale, ranging from -3 to +3, corresponding to very cold and very hot and 0 being the thermal neutral condition) |

| Reference | A | n | C | TE | LE | AE | IAQ | CAT | Studied variable | Applied method |
| --- | --- | --- | --- | --- | --- | --- | --- | --- | --- | --- |
| Gentile et al. | ✓ | 72 | 5 | RH_i_, t_a_ | E_c_, E_amb_, L_c_, L_w_, CRI, CCT, SPD, U_0_ |  | CO_2_ | PLC | Perception of lighting | Perceived Outdoor Lighting Qualities (POLQ) and has been successfully used in other indoor lighting studies: glaring from the light fixtures, glaring from the window, direct sunlight on your working space, flicker from the light fixtures, lighting quality |
|  |  |  |  |  |  |  |  | ER | Positive en negative emotions | Positive and negative affect scales (PANAS) |
|  |  |  |  |  |  |  |  | ER | Basic emotions | Basic emotional process scale (BEPS) |
|  |  |  |  |  |  |  |  | PR | Health | salvia cortisol concentration |
| Granito & Santana | n/ap |  |  |  |  |  |  | PTC | Temperature | Focus group discussion |
|  |  |  |  |  |  |  |  | PLC | Artificial room light Natural light | Focus group discussion |
|  |  |  |  |  |  |  |  | PAC | Acoustics | Focus group discussion |
| Hoque et al. | ✓ | 409 | 1 | t_a_, t_o_, RH_i_, RH_o_, v_a_ |  |  |  | PTC | Thermal comfort | Self-reported sensation |
|  |  |  |  |  |  |  |  | AP | Academic performance | Student exam scores |
| Jaakkola | ✓ |  |  |  |  |  |  | PR | Skin | Listing related health problems: itchy skin, skin irritation, skin rash, dermatological skin problem |
|  |  |  |  |  |  |  |  | PR | Eye | Listing related health problems: itchy eyes, eye irritation, dry eyes |
|  |  |  |  |  |  |  |  | PR | Respiratory tract (upper respiratory symptoms) | Listing related health problems: dry throat, nasal dryness, nose irritation |
|  |  |  |  |  |  |  |  | PR | Central nervous system (Neural behavioural symptoms) | Listing related health problems: headaches, nausea, lethargy |
|  |  |  |  |  |  |  |  | PR | Mucositis | Listing related health problems: mucosal symptoms |
| Jaber et al. | ✓ | 499 | 9 | t_a_, t_r_, v_a_^=^, RH_i_^=^, | E_amb_^=^ | L_Aeq_^=^ | CO_2_ | PTC | Thermal comfort | Self-reported sensation |
|  |  |  |  |  |  |  |  | ER | Fatigue | Self-report of level |
|  |  |  |  |  |  |  |  | CR | Accuracy in vigilance tasks, memory tasks, complex tasks | BARS battery “behavioural assessment and research system” |
|  |  |  |  |  |  |  |  | PR | Central nervous system (Neural behavioural symptoms) | Self-report headaches |

| Reference | A | n | C | TE | LE | AE | IAQ | CAT | Studied variable | Applied method |
| --- | --- | --- | --- | --- | --- | --- | --- | --- | --- | --- |
| Jamaludin et al. | ✓ | 20 | 1 | RH_i_, t_a_ | E_amb_ | L_Aeq_ | CO_2_, TVOC | PIAQ | Perceived air quality | Perception of stuffy air |
|  |  |  |  |  |  |  |  | PTC | Thermal condition | Students' satisfaction level |
|  |  |  |  |  |  |  |  | PLC | Lighting condition | Perceived lighting level |
|  |  |  |  |  |  |  |  | PR | Skin | Self-reported dry skin |
|  |  |  |  |  |  |  |  | PR | Eye | Self-reported itchy eyes, tired eyes, blurred vision |
|  |  |  |  |  |  |  |  | PR | Respiratory tract (upper respiratory symptoms) | Self-reported sore throat, running nose, cough, breathing difficulties |
|  |  |  |  |  |  |  |  | PR | Central nervous system (Neural behavioural symptoms) | Self-reported headaches, dizzy |
|  |  |  |  |  |  |  |  | PQL | Learning quality | Perceived learning productivity |
| Jonsdottir | ✓ | 791 | 1 |  |  | F_O_, SPL |  | PAC | Ability to hear teacher voice | Self-report of effect amplification of teachers' voice in classrooms |
|  |  |  |  |  |  |  |  | ER | Fatigue | Self-report of prevalence of fatigue using a five-point rating scale |
|  |  |  |  |  |  |  |  | CR | Subjective attention | Self-report of teachers regarding students paying attention using a five-point rating scale |
|  |  |  |  |  |  |  |  | CR | Subjective concentration | Self-report of students regarding easiness to concentrate on lessons using a five-point rating scale |
| Kennedy | n/a |  |  |  |  |  |  | PLC | Illumination | Window selection and placement |
| Kuru and Calis | ✓ | 235 | 1 | t_a_, t_r_, RH_i_, v_a_ |  |  |  | PTC | Thermal comfort | Self-reported thermal acceptability (yes=1, no=0), air velocity (low=-1, neither low nor high=0, high=1) |
| Lamb and Shraiky | n/a |  |  |  |  |  |  |  | Design concepts |  |
| Lee et al. | ✓ | 312 | 3 | t_a_, t_r_, t_op_, RH_i_ | E_hor_ | L_Aeq_, SPL | CO_2_ | PIAQ | Indoor air quality | Self-assessment on a 7-point scale and acceptance (yes-no) |
|  |  |  |  |  |  |  |  | PTC | Thermal comfort | Self-reported sensation |
|  |  |  |  |  |  |  |  | PLC | Visual environment | Self-assessment on a 7-point scale and acceptance (yes-no) |
|  |  |  |  |  |  |  |  | PAC | Aural environment | Self-assessment on a 7-point scale and acceptance (yes-no) |
|  |  |  |  |  |  |  |  | PQL | Perceived learning performance | Self-reported performance scores for calculating, reading, understanding and typing |

| Reference | A | n | C | TE | LE | AE | IAQ | CAT | Studied variable | Applied method |
| --- | --- | --- | --- | --- | --- | --- | --- | --- | --- | --- |
| Madbouly et al. | ✓ | 5325 | 1 |  |  |  |  | PAC | Noise disturbance | Noises from inside and outside the classroom |
|  |  |  |  |  |  |  |  | PAC | Importance of IEQ factors | Self-reported importance of acoustics properties (listening environment) and echo (comfortable, confusing, echoes, clear, irritating, relaxing, other) |
|  |  |  |  |  |  |  |  | PIAQ | Importance of IEQ factors | Self-reported importance of ventilation |
|  |  |  |  |  |  |  |  | PLC | Importance of IEQ factors | Self-reported importance of lighting |
| Majewski et al. | ✓ | 101 | 2 | CLO, t_a_, t_o_, t_w_, v_a_ |  |  | CO_2_ | PTC | Thermal comfort | Self-reported thermal sensation (Fanger 7-points scale, ranging from -3 to +3, corresponding to very cold and very hot and 0 being the thermal neutral condition) |
| Markides | n/ap |  |  |  |  | L_Aeq_ |  | AP | Academic performance | The lip-reading test was used. A video (in black and white) and was presented to students through a recorder and monitor. Students' ability to read lips was scored |
| Maxwell | n/a |  |  |  |  |  |  |  | Noise |  |
| McDonald | ✓ | 78 | 4 |  |  | L_Aeq_ |  | AP | Transfer of academic information | Academic information was presented with a 5-minute videotape developed specifically for the study and knowledge. Next, immediately after watching the video, participants were instructed to write out their food intake during the past 24 hours on a blank sheet of paper. This task required the information learned while watching the videotape to be transferred into long-term memory. Finally, the participants were tested using 13 multiple-choice questions addressing the information presented in the videotape |
| Mishra et al. | ✓ | 348 | 3 | RH_i_, t_a_, t_g_, t_o_, v_a_ |  |  | CO_2_ | PTC | Thermal adaptation | Self-reported sensation |
|  |  |  |  |  |  |  |  | PTC | Clothing insulation value | Reported clothing (top, shirt, long-sleeve shirt, sweater/blazer, dress, skirt, jacket, open shoes, shoes, trouser, boots, scarf) |

| Reference | A | n | C | TE | LE | AE | IAQ | CAT | Studied variable | Applied method |
| --- | --- | --- | --- | --- | --- | --- | --- | --- | --- | --- |
| Mongkolsawat et al. | ✓ | 673 | 1 | RH_i_, RH_o_, t_a_, t_o_, v_a_ |  |  |  | PIAQ | Air freshness (including odour) | Self-reported on a scale from 1 (very uncomfortable) to 5 (very comfortable). |
|  |  |  |  |  |  |  |  | PTC | Thermal comfort | Self-reported on a scale from 1 (very uncomfortable) to 5 (very comfortable), acceptability (five-point ordinal scale) |
|  |  |  |  |  |  |  |  | PLC | Visual comfort | Self-reported on a scale from 1 (very uncomfortable) to 5 (very comfortable). |
|  |  |  |  |  |  |  |  | PAC | Hearing comfort | Self-reported on a scale from 1 (very uncomfortable) to 5 (very comfortable). |
|  |  |  |  |  |  |  |  | ER | Sleepiness | Perception of freshness (as opposed to sleepiness) using a five-point rating scale |
|  |  |  |  |  |  |  |  | CR | Subjective alertness | Perception of alertness using a five-point rating scale (from 1 – much lower than average to 5 – much higher than average) |
|  |  |  |  |  |  |  |  | CR | Subjective attention | Perception of how the IEQ affected their attention using a five-point rating scale |
|  |  |  |  |  |  |  |  | PQL | Perceived learning performance | Perception of how the IEQ affected their overall learning performance using a five-point rating scale |
| Nico et al. | ✓ | 126 | 1 | t_a_, t_g_, RH_i_, v_a_ |  |  |  | PTC | Thermal Preference Index | Self-reported willingness to change, unacceptability of thermal environment, unacceptability of air movement, thermal sensation (Fanger 7-points scale, ranging from -3 to +3, corresponding to very cold and very hot and 0 being the thermal neutral condition) |
| Norbäck et al. | ✓ | 232 | 2 | RH_i_, t_o_ |  |  | AC, AER, CO_2_, N_2_O, NCHO, PM_10_, PSV, VB, VM | PIAQ | Air quality | Self-reported air quality (extremely poor (0)–extremely good (6)) when entering the classroom (the first 15 min and the last hour), and quality of odour (no odour (0)–extremely strong odour (6)) |
|  |  |  |  |  |  |  |  | PTC | Thermal comfort | Room temperature (too cold (0)–too hot (6)), air humidity (extremely dry (0)–extremely humid (6)), air movement (draught) (no movement (0)–extremely draughty (6)) |
|  |  |  |  |  |  |  |  | PLC | Visual comfort | illumination (very good (0)–very poor (6)) |
|  |  |  |  |  |  |  |  | PAC | Noise disturbance | Noise in general and noise from ventilation system (no disturbing noise (0)–very disturbing (6)) |
|  |  |  |  |  |  |  |  | PR | Eye | Self-reported eye symptoms, using a six-point rating scale |
|  |  |  |  |  |  |  |  | PR | Skin | Self-reported dermal symptoms, using a six-point rating scale |
|  |  |  |  |  |  |  |  | PR | Respiratory tract (upper respiratory symptoms) | Self-reported sore throat, sinusitis ●, and nasal symptoms and breathing difficulties, using a six-point rating scale |
|  |  |  |  |  |  |  |  | PR | Central nervous system (Neural behavioural symptoms) | Self-reported headaches and nausea, using a six-point rating scale |
|  |  |  |  |  |  |  |  | ER | Tiredness | Self-reported tiredness, using a six-point rating scale |
| Reference | A | n | C | TE | LE | AE | IAQ | CAT | Studied variable | Applied method |
| Persinger et al. | ✓ | 21 | 4 |  |  | L_Aeq_ |  | ER | Fatigue | Self-report rating scale using anchors of 1 through 7 |
|  |  |  |  |  |  |  |  | CR | Subjective concentration | Self-report rating scale for concentration using anchors of 1 through 7 |
| Ramprasad and Subbaiyan | ✓ | 1295 | 1 | t_a_, t_g_, t_op_, v_a_ |  |  |  | PTC | Thermal environment | Satisfaction with temperature (1 = very dissatisfied, 5 = very satisfied), temperature acceptability (yes-no), satisfaction with air movement (1=more air movement, 2=no change, 3=less air), satisfaction with freedom to control the speed of ceiling fans, satisfaction with freedom to switch the ceiling fans, satisfaction with freedom to open/close the window/shutters |
|  |  |  |  |  |  |  |  | PIAQ | Indoor air quality | Satisfaction with air quality, freshness of air (1=very stale, 5=very fresh), air quality acceptability |
|  |  |  |  |  |  |  |  | PLC | Visual environment | Satisfaction with visibility to see the chalkboard/projector screen, satisfaction with daylight, daylight preference (1=brighter, 2=no change, 3=less brighter), satisfaction with freedom to switch the fluorescent lamps on/off |
|  |  |  |  |  |  |  |  | PAC | Acoustic environment | Satisfaction with acoustics, acoustics acceptability (1=very dissatisfied 5=very satisfied) |
|  |  |  |  |  |  |  |  | PQL | Perceived academic performance | Self-reported overall academic satisfaction, academic ambience and academic performance |
| Rouag-Saffidine and Benharkat | ✓ | 36 | 1 |  | E_ver_, DF, DFC |  |  | PLC | Perceived visual comfort | Do you appreciate daylight in your workplace? (answers on a 4 rating scale), do you assess indoor daylight as sufficient in summer/in winter? (answers on a 4/5 rating scale), do you experience incident sunlight upon your work-plane? (answers on a 3 rating scale), are you keen for some solar controls in this work place? (answer by Yes/No) |
| Sarbu and Pacurar | ✓ | 200 | 2 | RH_i_, t_a_, t_g_, t_r_, v_a_, |  |  | CO_2_ | PTC | Thermal comfort | Self-reported sensation |
|  |  |  |  |  |  |  |  | CR | Concentrated attention | Kraepelin test; pairs of numbers have to be compared and calculations have to be performed based on the outcome of the comparison |
|  |  |  |  |  |  |  |  | CR | Distributive attention | Prague test; ordering and comparing figures with a model figure |
| Shelton et al. | ✓ | 158 73 33 27 | 4 3 1 1 |  |  | L_Aeq_ |  | CR | Level of attention | Lexical decision task; four different types of were pairs presented: 1) word, word; 2) non-word, non-word; 3) word, non-word; and 4) non-word, word. Subjects were asked to respond as quickly as possible; they had to press 1 on the keyboard if both stimuli were words and to press 2 otherwise |
|  |  |  |  |  |  |  |  | AP | Academic performance | Academic information during class was discussed, the transferred knowledge was tested with 6 multiple-choice questions (with 4 answer options) and 2 short-answer questions |
| Reference | A | n | C | TE | LE | AE | IAQ | CAT | Studied variable | Applied method |
| Siqueira et al. | ✓ | 28 | 3 | RH_i_, t_a_, t_g_, t_r_, t_wb_, v_a_, p_a_ |  |  |  | CR | Accuracy in reasoning tasks | The abstract reasoning, verbal reasoning, numerical reasoning, spatial reasoning and the mechanical reasoning test |
|  |  |  |  |  |  |  |  | PR | Health | Measurement of heart rate and blood pressure |
| Valavanidis and Vatista | ✓ | 1004 | 1 | RH_i_, t_a_ |  | L_Aeq_ | Cl_2_, CO, CO_2_, HCHO, SO_2_, NO_2_, O_3_, SD, TSP, TVOC | PIAQ | Indoor air quality | Self-reported quality of air and smells |
|  |  |  |  |  |  |  |  | PR | Central nervous system (Neural behavioural symptoms) | Self-reported heavy-headed, headaches, dizziness (Yes, often”, “Yes, sometimes” and “No, never”; experience of the past 2 months) |
|  |  |  |  |  |  |  |  | PR | Eye | Self-reported burning or irritation of the eyes |
|  |  |  |  |  |  |  |  | PR | Respiratory tract (upper respiratory symptoms) | Self-reported sore throat |
| Van Someren et al. | n/a |  |  |  |  |  |  | PLC | Lighting controls |  |
| Witkowska and Gladyszews-Fiedoruk | ✓ | 30 | 1 | RH_i_,t_a_ |  | L_Aeq_ |  | PTC | thermal comfort | Self-reported (scale 1-glad, 2-neutral, 3-dissatisfied), humidity and air temperature (scale 1-very dissatisfied, 2-dissatisfied, 3-no opinion, 4-glad, 5- very glad), air temperature (scale 1-too hot, 2-warm, 3-ok, 4-cold, 5-too cold), degree of humidity (scale 1-too high, 2-high, 3-ok, 4-dry, 5-too dry) |
|  |  |  |  |  |  |  |  | PAC | Acoustic comfort | noise level (scale 1-very dissatisfied, 2-dissatisfied, 3-no opinion, 4-glad, 5- very glad), noise level (scale 1-too loud, 2-loud, 3-ok, 4-quietly, 5-too quiet) |
| Xiong et al. | ✓ | 10 | 36 | t_a_ | E_amb_ | SPL |  | CR | Level of attention | Number searching test; numbers 0 through 99 were sequenced out of order on papers. Subjects were asked to search 15 designated numbers in normal order from these 100 numbers |
|  |  |  |  |  |  |  |  | CR | Recognition of meaningless images | 10 meaningless images on paper were presented for 10 seconds. When time was up, the subjects were asked to pick them out from all 20 meaningless images on another paper as quickly as possible |
|  |  |  |  |  |  |  |  | CR | Perception-oriented task | The Rochester colour word test; 15 words of colours in another colour were presented on papers. Subjects were asked to pick out the word itself or its colour sequentially |
|  |  |  |  |  |  |  |  | CR | Problem-solving task | Reading comprehension; subjects were asked to pick out the only correct answer from multiple choices based on their own understandings. Previously printed out, five independent questions were randomly distributed to each subject from the administrative ability tasks for national civil servant selections |
| Yan et al. | n/ap |  |  | RH_i_, t_a_ | CRI, , CT, LF, E_amb_ |  | CO_2_ | CR | Visual performance | Recognition ability of real objects in a controlled environment |

| Reference | A | n | C | TE | LE | AE | IAQ | CAT | Studied variable | Applied method |
| --- | --- | --- | --- | --- | --- | --- | --- | --- | --- | --- |
| Yang and Becerik-Gerber | ✓ | 627 | 1 |  |  |  |  | PTC | Temperature perception | Self-reported discomfort, heat from the sun, heat from classroom equipment, cold air from windows, cold air from ac unit or vent, cold air from door or outside room source, noticeably different temperature than other classrooms of hallways |
|  |  |  |  |  |  |  |  | PIAQ | Air quality perception | Self-reported comfort, dirty air, humid air, dry air, odorous air, drafty air, stuffy air |
|  |  |  |  |  |  |  |  | PLC | Artificial lighting perception | Self-reported adequate illumination, too bright, too dark, too much glare, lack of control, undesirable colour, shadows, flickering |
|  |  |  |  |  |  |  |  | PLC | Daylight perception | Self-reported adequate illumination, too bright, too dark, too much sunlight, lack of control, shadows |
|  |  |  |  |  |  |  |  | PAC | Acoustics perception | Self-reported noise, from air vent/AC, from electronic equipment, from talking inside the classroom, from talking outside of the classroom |
| You et al. | ✓ | 10005 |  | RH_i_, t_a_, t_o_ |  |  | CO_2_ | PIAQ | Indoor air quality | Self-reported evaluation of how ventilation rate affected comfortlessness |
| Zomorodian et al. | n/ap |  |  |  |  |  |  | PTC |  | Study reports application of thermal sensation (seven-point ASHRAE scale and Bedford scale), thermal preference (McIntyre three-point rating scale and checklist for clothing and activity |

Notes: A=Applicability; ✓=method is applied for systematic approach; n=number of participants; C=number of campaigns, trials or runs; n/a=information about the applied method is not available; n/ap=information about the method is not applicable for quantitative research; = condition was kept constant during campaign(s)

TE=Thermal environment: RH_i_=indoor relative humidity; RH_o_=outdoor relative humidity; t_a_=air temperature; t_f_=floor temperature; t_g_=globe temperature; t_o_=outdoor temperature; t_op_=operative temperature; t_r_=radiant temperature; t_r___asym_=radiant asymmetry temperature; t_w_=temperature of walls; t_wb_=wet-bulb temperature; v_a_=air velocity

LE=Lighting environment; Cc=contrast; CCT=correlated colour temperature; CRI=colour rendering index; CT=colour temperature; DF=daylight factor; DFC=daylight factor contour; E_amb_=ambient illuminance; Ec=cylindrical illuminance; E_hor_=horizontal illuminance; E_ver_=vertical illuminance; L_c_=average ceiling luminance; L_f_=luminous flux; L_og_Ch=chloropic lux; L_og_Cy=cyanopic lux; L_og_Er=erythropic lux; L_og_Ph=photopic lux; L_og_Rh=rhodopic lux; L_w_=average wall luminance; SPD =spectral power distribution; U0 =illuminance uniformity

AE=Acoustic environment: L_Aeq_=background noise or ambient noise; F0=fundamental frequency; RT=reverberation time; SPL_a_ =A-weighted sound pressure level; SPL_c_ =C-weighted sound pressure level

IAQ=Indoor air quality; AC=allergen concentration; AER=air exchange rate; Cl_2_=chlorine; CO=carbon monoxide; CO_2_=carbon dioxide CO_2_o=carbon dioxide outside; D=dust; HCHO=formaldehyde; NO_2_=nitrogen dioxide; O_3_=ozone; PM10=particles; PSV=personal supply ventilation; SD=settled dust; SO2=sulphur dioxide; TSP=total respirable suspended particulate matter; TVOC=total volatile organic compounds; VB=viable bacteria; VM=viable mouts

CAT=studied category; AP=academic performance; CR=cognitive response; ER=Emotional response; PIAQ=perceived indoor air quality; PLC=perceived lighting comfort; PR=physical response; PTC=perceived thermal comfort;

^1^ Number of university students

^2^ During 5 days participants filled in daily heat-health symptom log at each hour of the day between 8 a.m. and 2 p.m.

^3^ All experiments were carried out on weekday evenings (Monday till Thursday, 6:30-10:00 p.m.) in a 3-month period

^4^ Study reports that more than 100 questionnaires were distributed

^5^ Study reports that questionnaire was distributed to 1000 undergraduate students

**APPENDIX C** Overview of the PANAS and BEPS method, the Karolinska Sleepiness Scale and the Pittsburgh Sleep Quality Index

| **Method** | **Included** | **Score range** | **Dimension** | **Abbreviation** | **Items** |
| --- | --- | --- | --- | --- | --- |
| PANAS | Yes | 10–50 | Positive affect scale | PA | Interested + excited + strong + enthusiastic + proud + alert + inspired + determined + attentive + active |
|  |  | 10–50 | Negative affect scale | NA | Distressed + upset + guilty + scared + hostile + irritable + ashamed + nervous + jittery + afraid |
| BEPS | Yes | 1–4 | Activation | BEPSA | Average (rested - drowsy + awake) |
|  |  | 1–4 | Evaluation | BEPSE | Average (friendly - sad - anxious) |
|  |  | 1–4 | Orientation | BEPSO | Average (interested - quiescent + engaged) |
|  |  | 1–4 | Control | BEPSC | Average (independent - indecisive - weak) |
|  |  | 1–4 | Emotional status | BEPSES | Average (Activation + Evaluation + Orientation + Control) |
| Karolinska Sleepiness Scale | Yes | 1-10 | Sleepiness versus alertness | KSS | Scale from (1) Extremely alert to (10) great effort to keep awake, fighting sleep |
| Pittsburgh Sleep Quality Index | No | 0-3 | Sleep quality | PSQI | Subjective sleep quality, sleep latency, sleep duration, habitual sleep efficiency, sleep disturbances, use of sleeping medication, daytime dysfunction over a long period of time |

Notes: BEPS=basic emotional process scale; BEPSA=BEPS activation; BEPSC=BEPS control; BEPSE= BEPS evaluation; BEPES=BEPS emotional status; BEPSO=BEPS orientation; KSS=Karolinska sleepiness scale; PSQI=Pittsburgh sleep quality index

**APPENDIX D** Measured indoor environmental parameters, symbol and description of the measuring device.

| **Performance indicator** | **Symbol** | **Description** |
| --- | --- | --- |
| Outdoor air temperature | t_o_ | The outside temperature was derived from the Royal Netherlands Meteorological Institute, [www.knmi.nl](http://www.knmi.nl), reading interval 1 hr |
| Indoor air temperature at desktop height | t_a_ | Air temperature in degrees Celsius (°C) and is measured with an ATAL VLK-60W temperature sensor at 1.1 m height, accuracy ±0.5 °C @ 0 to +50 °C |
| Outdoor relative humidity | RH_o_ | The outdoor relative humidity was derived from the Royal Netherlands Meteorological Institute, [www.knmi.nl](http://www.knmi.nl), reading interval 1 hr |
| Indoor relative humidity | RH_i_ | Indoor relative humidity in percentage (%) and is measured with an ATAL VLK-60W humidity sensor at 1.1 m height, accuracy, accuracy ±0.3 % RH_i_ @ 5 to 99 % RH_i_ |
| Background noise | BGN | Average sound pressure level in dB(A) over a period of 45 seconds and is measured with a Velleman DEM201, accuracy +/- 1.4 dB 94 dB @ 1 kHz |
| Carbon dioxide concentration | CO_2_ | Parts per million carbon dioxide concentration (ppm CO2) is measured with an ATAL VLK-60W carbon dioxide sensor at 1.1 m height, accuracy ±75 ppm + 10% of the actual reading |
| Particulate matter 10 | PM10 | Particulate matter is measured with an ATAL VLK-60W PM10 sensor at 1.1 m height sensor: accuracy < ±15% @ 0 to 1,000 μg/m^3^ |
| Particulate matter 2.5 | PM2.5 | Particulate matter is measured with an ATAL VLK-60W PM2.5 sensor at 1.1 m height sensor: accuracy < ±15% @ 0 to 1,000 μg/m^3^ |
| Horizontal illuminance | E_hor_ | Illuminance level in Lux and is measured with a VOLTCRAFT MS-1300, accuracy ± 5% + 10 digits @ < 10.000 lux |
| Volatile organic compounds | TVOC | Volatile organic compounds is measured with an ATAL VLK-60W TVOC sensor at 1.1 m height sensor: accuracy ± 0.02 mg (or 10%) @ 0 to 3.5mg/m^3^ |

**APPENDIX E** Assumed linear relation between variables

| **KPI** | **Variable** | **Assumption of linearity** | **Assumed association** | **Remarks** |
| --- | --- | --- | --- | --- |
| CO_2_  PM2.5  TVOC | PIAQ | An increase in all KPI’s will lead to a deterioration in the perceived indoor air quality (PIAQ) | Negative correlation | PIAQ score, minimum score is 1= very poor, and the maximum score is 5= very good. Optimum is maximum score of scale, linear analyses is possible |
| RH_i_  t_a_ | PTC_sens_ | Increase of RH_i_ and T_a_ will lead to an increase of the thermal sensation vote (PTC_sens_) | Positive correlation | Optimum is 4 on a scale from 1 to 7. Although al linear correlation is assumed between the KPI’s and the PTC_sen,_ the optimum of this scale (neutral sensation) is in the middle (4) and prohibit further linear analyses. For linear analyses, this variable is recoded into the level of perceived thermal comfort (PTC) due to cold or heat. |
| RH_i_  t_a_ | PTC_pref_ | Increase of RH_i_ and T_a_ will lead to an increase of the thermal preference vote (PTC_pref_) | Positive correlation | Optimum is 4 on a scale from 1 to 7. Although al linear correlation is assumed between the KPI’s and the PTCpref, the optimum of this scale (Neither warmer nor colder) is in the middle (4) and prohibit further linear analyses. For linear analyses, this variable is recoded into the level of perceived thermal comfort based on the thermal preference warmer or colder. |
| E_hor_ | PLC | Increase of amount of lux will lead to an increase of the perceived lighting comfort (PLC) | Positive correlation | PLC score, minimum score is 1= very poor, and the maximum score is 5= very good. Optimum is maximum score of scale, linear analyses is possible |
| RT | PAC | Increase of the RT will lead to a deterioration of the perceived acoustic comfort (PAC) | Negative correlation | PLC score, minimum score is 1= very poor, and the maximum score is 5= very good. Optimum is maximum score of scale, linear analyses is possible |

^63^
